# Placenta-derived extracellular vesicles from preeclamptic and healthy pregnancies impair *ex vivo* vascular endothelial function

**DOI:** 10.1042/BSR20222185

**Published:** 2022-12-16

**Authors:** Roberto Villalobos-Labra, Ricky Liu, Floor Spaans, Tamara Sáez, Anita Quon, Michael Wong, Desmond Pink, John Lewis, Manu Vatish, Sandra T. Davidge, Christy-Lynn M. Cooke

**Affiliations:** 1 Department of Obstetrics and Gynecology, Edmonton, Alberta, Canada; 2 Women and Children’s Health Research Institute, Edmonton, Alberta, Canada; 3 Department of Physiology, Edmonton, Alberta, Canada; 4 Department of Oncology, University of Alberta, Edmonton, Alberta, Canada; 5 Nanostics Inc., Edmonton, Alberta, Canada; 6 Nuffield Department of Women’s and Reproductive Health, University of Oxford, Oxford, U.K.

**Keywords:** endothelial dysfunction, extracellular vesicles, placenta, preeclampsia, vascular function

## Abstract

Preeclampsia (PE) is a pregnancy syndrome characterized by new-onset hypertension and end-organ dysfunction. The pathophysiology of PE remains undetermined, but it is thought that maternal vascular dysfunction plays a central role, potentially due, in part, to the release of syncytiotrophoblast-derived extracellular vesicles (STBEVs) into the maternal circulation by a dysfunctional placenta. STBEVs from normal pregnancies (NP) impair vascular function, but the effect of PE STBEVs (known to differ in composition with elevated circulating levels) on vascular function are not known. We hypothesized that PE STBEVs have more detrimental effects on vascular function compared with NP STBEVs. STBEVs were collected by perfusion of placentas from women with NP or PE. Mesenteric arteries from pregnant rats were incubated overnight with NP or PE STBEVs, and vascular function was assessed by wire myography. NP and PE STBEVs impaired endothelial function, partially by reducing nitric oxide (NO) bioavailability. Incubation of human umbilical vein endothelial cells with NP and PE STBEVs increased nuclear factor κ-light-chain-enhancer of activated B cell (NF-κB) activation, reactive oxygen species, nitrotyrosine levels, and reduced NO levels. However, PE STBEVs increased NF-κB activation and nitrotyrosine levels to a lesser extent than NP STBEVs. Taken together, no greater impact of PE STBEVs compared with NP STBEVs on endothelial function was found. However, the impaired vascular function by PE STBEVs and increased levels of STBEVs in PE suggest PE STBEVs may contribute to maternal vascular dysfunction in PE. Our study further expands on the potential mechanisms that lead to adverse outcomes in PE and provides potential targets for future interventions.

## Introduction

Preeclampsia (PE) is a pregnancy disorder occurring in 5–7% of pregnancies worldwide [1]. It is a major cause of maternal and neonatal mortality and a risk factor for later-life cardiovascular disease in the mother and offspring [2]. PE is characterized by new-onset hypertension with proteinuria and/or end-organ dysfunction after 20 weeks of gestation [3]. The pathophysiology of PE is believed to originate from a dysfunctional placenta and stress-associated placental-derived factors released into the maternal circulation [4]. The central role of the placenta in this syndrome is illustrated by the fact that the symptoms of PE are resolved only after delivery [5]. The placenta-derived circulating factors are thought to cause maternal vascular endothelial cell dysfunction [6,7], a hallmark of PE and one of the primary causes of maternal complications in this syndrome [8]. Women with PE show impaired endothelium-dependent vasodilation, primarily due to reduced nitric oxide (NO) bioavailability (reviewed in [9]). NO is one of the main endothelium-released vasodilator molecules and essential for normal vascular function in pregnancy [10,11]. The reduction in NO bioavailability in the vasculature of women with PE is largely due to increased levels of reactive oxygen species (ROS), in particular, superoxide radicals [12]. Moreover, the reaction between NO and superoxide results in the formation of the potent oxidant peroxynitrite, which further impairs endothelial function by inducing nitrative stress [13,14].

While maternal vascular dysfunction in PE is widely described, the causal role of the placenta is still unclear [15]. There is growing evidence suggesting that syncytiotrophoblast-derived extracellular vesicles (STBEVs) may be a crucial link between the placenta and the maternal vasculature [6]. Various studies have reported higher maternal peripheral plasma levels of STBEVs in PE compared with normal pregnancies [16–19]. In addition, STBEVs from women with normal pregnancies (NP STBEVs) were shown to impair endothelium-dependent vasodilation in subcutaneous fat arteries from pregnant women [20] as well as in uterine arteries from pregnant rats [21] and mesenteric arteries from pregnant mice [22]. Moreover, NP STBEVs have been shown to disrupt the endothelial cell monolayer *in vitro* [23] and induce nitrative stress in endothelial cells [24]. However, recent literature evaluating STBEVs from women with PE shows that PE STBEVs have a different protein [25,26], lipid [27] and miRNA [28] composition, suggesting they may have different biological effects in comparison with NP STBEVs. For instance, PE STBEVs, but not NP STBEVs, were reported to increase monocyte adhesion to endothelial cells [29], a process that is associated with endothelial dysfunction [30]. Moreover, in immune cells, PE STBEVs were shown to activate nuclear factor κ-light-chain-enhancer of activated B cells (NF-κB), a transcription factor that reduces endothelium NO synthase expression [31] and promotes ROS production through increasing nicotinamide adenine dinucleotide phosphate oxidase (NADPH oxidase) expression [32]. However, the mechanisms by which PE STBEVs affect vascular endothelial cell function, and whether they are distinct from NP STBEVs, have not been explored.

In the current study, we hypothesized that PE STBEVs have a more detrimental effect on vascular function compared to NP STBEVs. To address our hypothesis, we assessed (1) the impact of PE compared with NP STBEVs on vascular function of mesenteric arteries from pregnant rats by wire myography *ex vivo* and examined the potential mechanisms by assessing (2) the effects of PE compared with NP STBEVs on NF-κB activation, ROS, nitrative stress and NO levels using isolated human umbilical vein endothelial cells (HUVECs) *in vitro*.

## Methodology

### Clinical samples

Informed consent was obtained from all mothers undergoing elective cesarean section whose tissues (placentas and umbilical cords) were used in the present study. Umbilical cords were collected from placentas of uncomplicated pregnancies immediately after delivery and transported in phosphate-buffered saline (PBS) from the Lois Hole Hospital for Women (LHHW)/Royal Alexandra Hospital to our laboratory at the University of Alberta. Patients with PE were recruited according to the diagnostic criteria defined by the International Society for the Study of Hypertension in Pregnancy (ISSHP) [33,34]. PE was defined as *de novo* hypertension in the second half of pregnancy (>140/90 mmHg, on at least two occasions) along with proteinuria (+2-+3 urine dipstick). The clinical characteristics of the NP (*n*=4) and PE (*n*=4) patients whose placentas were used are summarized in Table 1.

**Table 1:** Clinical data of the patients undergoing elective cesarean section that consented for STBEVs isolation

	NP (*n*=4)	PE (*n*=4)	*P* value
**Maternal age (years)**	27 ± 2 (26–30)	30±6 (22–37)	ns
**Gestation (Weeks)**	39.0 ± 0.5 (38.3–39.5)	37.1 ± 1.1 (35.6–38.4)	0.0210
**Highest systolic BP (mmHg)**	128 ± 3 (124–130)	172 ± 17 (153–195)	0.0023
**Highest diastolic BP (mmHg)**	75 ± 5 (70–81)	107 ± 7 (100–114)	0.0002
**Pre-pregnancy weight (kg)**	57.0 ± 8.6 (50.0–68.4)	88.8 ± 39.6 (54.4–128.0)	ns
**Height (cm)**	163 ± 11 (153–178)	158 ± 1 (157–160)	ns
**Pre-BMI (kg/m^2^)**	21.4 ± 1.4 (19.5–23.0)	35.4 ± 15.3 (22.1–50.0)	ns
**Proteinuria (N° +)**	NA	2-3	
**Urate (mmol/L)**	NA	346 ± 68 (270–404)	
**Hematrocrite (L/L)**	0.36 ± 0.01 (0.35–0.37)	0.35 ± 0.02 (0.33–0.38)	
**Newborn sex (F/M)**	2/2	1/3	
**Newborn weight (g)**	3018 ± 275 (2620–3230)	2763 ± 681.8 (1970–3520)	ns
**Smokers (n)**	0	1	

Data presented as mean ± SEM. Differences were considered significant when *P*<0.05; BMI, body mass index; BP, blood pressure; na, not assessed; NP, normal pregnancy; ns, not significant; PE, preeclampsia.

### Placental perfusion for collection of STBEVs

Isolation of STBEVs were conducted at the LHHW within 10–20 min of delivery. STBEVs were isolated using a dual placental lobe perfusion system, as previously described and developed by Dr Christopher Redman and Dr Ian Sargent’s group at Oxford University, U.K. [35,36]. In short, *ex vivo* reestablishment of fetal and maternal circulation in an intact placental lobe was performed. Filtered (0.1 µm, Nalgene Rapid-Flow Sterile Single Use Vacuum Filter, Thermo Scientific, Waltham, MA, U.S.A.) fetal media contained: 8 mg/ml Dextran (MilliporeSigma, Burlington, MA, U.S.A.), 5 mg/ml bovine serum albumin (BSA, MilliporeSigma), 5 IU/ml heparin (Sandoz Canada Inc., QC, Canada), 1% antibiotic–antimycotic solution (MilliporeSigma), and 100 IU/ml Alteplase (to dissolve blood clots; Cathflo, Roche, Basel, Switzerland). Filtered (0.1 µm) maternal media contained: 5 mg/ml BSA, 5 IU/ml heparin and 1% antibiotic–antimycotic solution. The fetal outflow was continuously monitored (4–5 ml per min) to ensure the integrity of the fetal circulation in the placental lobe, while STBEVs were brushed off and collected in the maternal outflow. After perfusion (2–3 h), the maternal media were centrifuged at 1500 ***g*** to remove any cells contained in the perfusate. The supernatant was ultracentrifuged at 150,000 ***g*** for 2 h in a Beckman Coulter Optima XE-90 Ultracentrifuge (Beckman Coulter Inc, Brea, California, U.S.A.), and the pellet was resuspended in filtered PBS (0.25 µm) and ultracentrifuged again at 150,000 ***g*** for 2 h. The final pellet (i.e. the STBEVs) was resuspended in 1–2 ml of PBS, aliquoted, and stored at −80°C until further use.

### Microflow cytometry

The percentage of EVs from placental origin were estimated via microflow cytometry using Apogee A60 MicroPlus Microflow cytometer (Apogee Flow Systems Ltd, Northwood, UK.). Information regarding the optimization, settings for microflow cytometry, the detailed protocol, as well as the Minimal Information for Studies of Extracellular Vesicles (MISEV) guidelines and MIFlowCyt-EV guidelines [37,38] are provided in Supplementary Tables 1 and 2 and Supplementary Material for instrument set-up and optimization. Experiments describing how the instrument was qualified for small particle analysis have been described [39]. The instrument was cleaned and calibrated daily prior to sample analysis using a variety of silica and polystyrene standards (Apogee Bead Mixtures [1524,1527]). Fluorescent calibration was achieved using MESF beads [Spherotech RCP-05]), particle size was qualified using polystyrene standards. Optimal antibody and membrane dye concentrations were defined in earlier experiments (Microflow cytometry supplementary figures). STBEVs preparations were diluted (1:200), incubated with antibody against placental alkaline phosphatase (PLAP) for 20 min (0.25 mg/ml, NDOG2 mouse antibody [17]), APC goat anti mouse for 20 min (0.1 mg/ml), carboxyfluorescein diacetate succinimidyl ester (CFDA-SE, used to stain EVs, Invitrogen, ThermoFisher), for 30 min at 37°C (6.25 µmol/L), and then diluted in PBS. CFDA-SE was used to stain EVs. CFDA-SE is permeable to EVs membrane and needs to be processed by esterases to convert CFDA-SE into the active (increased fluorescence) form carboxyfluorescein succinimidyl ester (CFSE). CFSE covalently binds to free amines on proteins inside EVs [40]. Since CFDA-SE needs to be processed by esterases to be converted into the fluorescent form CFSE, mainly EVs but not protein aggregates or other membranes are expected to be stained with CFSE [40]. Samples were assayed in triplicate, run for at least 60 s, and conventional manual gating analysis of FCM data was performed using Histogram version 6.049 software (Apogee Flow Systems).

### Nanoparticle Tracking Analysis

Particle size and concentration were measured by Nanoparticle Tracking Analysis (NTA) using a NanoSight LM10 system equipped with a 405 nm laser (NanoSight, Amesbury, U.K.) as described [41]. Instrument was checked to confirm optimal functionality prior to sample data collection using NIST traceable 125 nm-polystyrene beads (3000 series) (Thermo Scientific, Waltham, MA, U.S.A.) diluted 1:50,000 in filtered (100 nm) 10 mmol/L potassium chloride. The NIST specifications for the listed size of the 125 nm particles is 122 ± 3 nm; instrument data on the day of analysis yielded a mean size of 125 ± 9.2 nm.

EV samples were diluted 1:10,000 in PBS to achieve a concentration of 20–90 particles/frame and injected into the sample chamber with sterile syringes. Three replicates from each sample were measured, each run for 1 min. The temperature during sample acquisition was recorded to accurately determine particle concentration. All samples were captured with the camera level set at 11 and detection level threshold set at 5. The unit was cleaned between samples and instrument cleanliness was checked prior to starting using diluent.

### Presence of TSG101, PLAP and cytochrome *c* in/on STBEVs by Western blotting

To further confirm the presence and placental-specificity of EVs in our STBEVs preparations, the presence of Tumor susceptibility gene 101 (TSG101, marker of extracellular vesicles [37]), placental alkaline phosphatase (PLAP, placental marker, carried by extracellular vesicles released by the syncytiotrophoblast [35]), and cytochrome *c* (CytC, cell marker, negative control – not present in extracellular vesicles [37]) were determined using Western blotting. STBEV-samples and HUVECs from normal pregnancies (used as a positive control) were homogenized in RIPA buffer (50 mmol/L Tris-HCl, pH 7.4, 150 mmol/L NaCl, 1% Triton X-100 [Northwest Scientific, Billings, MT, U.S.A.], 0.5% sodium deoxycholate, 0.1% sodium dodecyl sulfate [SDS], 1 mmol/L EDTA, 10 mmol/L NaF, 1 mmol/L phenylmethylsulfonyl fluoride [PMSF, MilliporeSigma]), with protease inhibitor (Halt™ Protease Inhibitor Cocktail, ThermoFisher). Protein concentrations were determined by bicinchoninic acid assay (BCA, Pierce, Rockford, IL, U.S.A.). Then, 100 μg of protein was loaded and run on SDS polyacrylamide gels (10% for PLAP and TSG101, 12% for CytC). Proteins were transferred to nitrocellulose membranes (Biorad, Mississauga, ON, Canada), and total protein staining was performed using Revert™ total protein stain (LI-COR Biosciences, Lincoln, NE, U.S.A.). Membranes were incubated with Blocking Buffer for Fluorescent Western Blotting (Rockland Immunochemicals, Pottstown, PA, U.S.A.) and then incubated overnight (4°C) with one of the following primary antibodies rabbit polyclonal anti-TSG101 (1:1000, MilliporeSigma #T5701), mouse monoclonal anti-PLAP (1:1000, NDOG2, provided by Dr Manu Vatish’s laboratory; Oxford University, U.K.), and mouse monoclonal anti-CytC (1:1000, BD Biosciences, Franklin Lakes, New Jersey, U.S.A., #556433). The next day, membranes were incubated for 1 h with their corresponding secondary antibodies: IRDye™ 800RD-labeled donkey anti-mouse (against CytC primary antibody, 1:10.000; LI-COR Biosciences), IRDye™ 800RD-labeled donkey anti-rabbit (against TSG101 primary antibody, 1:10,000; LI-COR Biosciences), and IRDye™ 680RD-labeled donkey anti-mouse (against PLAP primary antibody, 1:10.000; LI-COR Biosciences). Protein bands were imaged using the LI-COR Odyssey Imaging Systems v3.0 (LI-COR Biosciences).

### Experimental design for ex vivo vascular function experiments

Female and male (for breeding) Sprague-Dawley (SD) rats were purchased at 3 months of age from Charles River, Canada (Saint-Constant, QC, Canada). The rats were housed under a standard day:night cycle (10:14 h) with *ad libitum* access to food and water. All rats were allowed at least 1 week of acclimatization after arrival and at the age of 3–4 months, female rats were mated by overnight housing with a male, and the presence of sperm in a vaginal smear was designated as gestational day (GD) 0. On GD 20, rats were killed by exsanguination under inhaled isoflurane anesthesia. Second-order mesenteric arteries were isolated, divided into 2 mm pieces (7–8 segments per rat, depending on the experimental design), and mounted on a single 40 µm wire. Mesenteric artery segments were then incubated with or without NP or PE STBEVs (10–200 μg/ml) in culture media (Medium 199 [M199, Gibco, ThermoFisher] supplemented with 8% Fetal Bovine Serum [FBS, MilliporeSigma], 1% penicillin-streptomycin [Gibco], and 0.1% gentamicin [MilliporeSigma]) for 16 h at 37°C. The maximum concentration (200 µg/ml) was chosen based on previous studies evaluating effects of NP STBEVs on vascular function [20,21,24,42].

### Vascular function by wire myography

After 16 h of incubation, mesenteric artery segments were mounted onto a wire myograph system (DMT, Copenhagen, Denmark) using 40 µm wires to assess vascular function. After mounting, vessels were allowed to equilibrate until stable in HEPES-buffered physiological saline solution (PSS; in mmol/L: 142 NaCl, 4.7 KCl, 1.17 MgSO_4_, 4.7 CaCl_2_, 1.18 K_2_PO_4_, 10 HEPES, 5.5 glucose, pH 7.4) and were then normalized (IC_100_ = 0.8; 13.3 kPa). Vessels were allowed to equilibrate for 20 min and then received a wakeup dose of 10 µmol/L phenylephrine (MilliporeSigma) followed by a dose (3 × 10^−6^ mol/L) of methylcholine (MCh, MilliporeSigma), an endothelium-dependent vasodilator, to test for endothelial integrity and function. Baths were washed three times with PSS and then incubated with or without the pan nitric oxide synthase inhibitor Nω-Nitro-L-arginine methyl ester hydrochloride (L-NAME, 100 µmol, MilliporeSigma) for 30 min. Arteries were pre-constricted with U46619 (thromboxane A2 analogue, MilliporeSigma) at a previously determined EC_80_ dose (1.6 × 10^−7^ mol/L; data not shown). Endothelium-dependent vasodilation responses were assessed with a cumulative concentration response curve to MCh (CCRC; 1 × 10^−9^ to 1 × 10^−4^ mol/L MCh; in 2 min intervals). Vessels were then washed three times with PSS and pre-constricted with U46619 to assess an endothelium-independent vasodilation using sodium nitroprusside (SNP) CCRC (NO donor, 1 × 10^−11^ to 1 × 10^−5^ mol/L, MilliporeSigma). Then, vessels were washed three times with PSS and each bath was incubated with a high potassium salt solution (KPSS; 123 mmol/L) to assess non-receptor-mediated vasoconstriction responses. Data were analyzed using LabChart software (ADInstruments; Colorado Springs, CO, U.S.A.), and the maximum effect (*E*_max_, computed as the point of maximal vasodilation for each *n*), area under the curve (AUC), and EC_50_ were calculated.

### Isolation of human umbilical vein endothelial cells (HUVECs)

HUVECs were isolated from the collected umbilical cords according to standard methods, as previously described [24,43]. After isolation, cells were seeded in a T25 flask and cultured in primary culture medium (PCM; Medium 199 supplemented with 20% FBS, 1% penicillin-streptomycin, 0.1% gentamicin, and 1% Endothelial Cell Growth Supplement [ECGS]-heparin [Corning, NY, U.S.A.]) until passage 2. At passage 3, cells were plated for the experiments described below.

### Assessment of NF-κB activation and nitrotyrosine levels in HUVECs by confocal laser scanning microscopy

To evaluate the effect of NP and PE STBEVs on markers of endothelial dysfunction [13,44], we assessed the activation of NF-κB and nitrotyrosine levels in HUVECs. HUVECs were seeded (16 × 10^4^ cells/well) at passage 3 on gelatin-coated (1% w/V) glass coverslips placed in 24 wells in PCM and were allowed to grow to 100% confluence, as previously described [45]. Cells were then cultured in PCM without ECGS and incubated with or without NP or PE STBEVs (100 µg/ml) for 6 h (to assess NF-κB translocation) or for 24 h (to assess nitrotyrosine levels). Cells were fixed in 4% paraformaldehyde, and then permeabilized and blocked with 1% BSA-containing blocking solution (50 mmol/L NH_4_Cl, and 0.05% Triton X-100 in PBS). NF-κB was immunolocalized by incubating the cells with a monoclonal antibody mouse anti-NF-κB p65 subunit (1:100, Santa Cruz #sc-8008), and nitrotyrosine levels were determined by incubation with a monoclonal antibody mouse anti-nitrotyrosine (1:100, Novus Biologicals #NB110-96877) overnight at 4°C in 1% BSA-blocking solution. The cells were then incubated with the secondary antibody goat anti-mouse Alexa Fluor™ 546 (1:300, ThermoFisher #A-11003) in 1% BSA-blocking solution. Nuclei were counterstained with DAPI (4′,6′-diamidino-2-phenylindole, 5 μmol/L, ThermoFisher) and sections were mounted with mounting medium containing DAPI (Vector Laboratories, Burlingame, CA, U.S.A.). Images were obtained using a confocal Zeiss LSM 700 microscope with Zen Black software (Zeiss, Toronto, ON, Canada). NF-κB and nitrotyrosine images were taken with 630× and 200× magnification, respectively. Images were analyzed using FIJI ImageJ 1.53n (Wayne Rasband NIH, U.S.A.). NF-κB nuclear fluorescence was evaluated in the region restricted to the nuclear area demarcated by DAPI fluorescence, and nitrotyrosine fluorescence intensity was determined by taking the fluorescence for the entire cell. Nitrotyrosine and NF-kB mean fluorescence intensity were presented as the fold change relative to the mean fluorescence intensity of the non-treated cells within the same experiment.

### ROS levels in HUVECs by DHE staining

An increase in oxidative stress is associated with endothelial dysfunction [14]. Thus, the impact of STBEVs on oxidative stress was evaluated by assessing ROS levels in HUVECs. Confluent HUVECs (passage 3) grown on 48-well plates were cultured in PCM without ECGS and incubated with or without NP or PE STBEVs (100 µg/ml) for 24 h. After this, the media was removed and cells were washed once with cold PBS. Diluted Dihydroethidium (DHE) (20 µM, Biotium, Burlington, Canada) was added to the cultures, and plates were incubated at 37°C for 30 min. The DHE solution was aspirated, cells were washed and pictures were immediately taken on an Olympus IX81Microscope (Olympus America INC, Melville, NY, U.S.A.) using TRITC wavelength. All pictures were taken at 10× magnification and were analyzed using FIJI ImageJ 1.53n [46]. The DHE fluorescence intensity was determined within the entire cells, and fluorescence intensity was presented as fold change relative to the mean fluorescence intensity of the non-treated cells within the same experiment.

### Assessment of intracellular nitric oxide in HUVECs

Since NO is crucial for proper endothelial function [47] and NO contribution to endothelium-dependent vasodilation was reduced in the vascular function experiments, the effects of NP or PE STBEVs on NO levels in HUVECs were evaluated. Intracellular NO levels were determined using the fluorescent dye DAF-FM diacetate (4-amino-5-methylamino-2',7'-difluorofluoresceind iacetate, Cayman Chemical, Ann Arbor, MI, U.S.A.), as previously described [48]. Confluent HUVECs (25,000 cells/well) at passage 3 were grown on 96-well black/clear bottom plates (Thermo Fisher Scientific) coated with gelatin 1% and cultured in PCM without ECGS. Cells were incubated with or without NP or PE STBEVs (100 µg/ml) for 24 h. The next day, HUVECs were incubated with the fluorescent dye DAF-FM diacetate in M199 without phenol red (Gibco) for 30 min, with or without the pan NOS inhibitor (100 μmol/L) L-NAME to evaluate the NOS-dependent intracellular NO production. Fluorescence (λ_ex_/λ_em_: 490/525 nm) was measured in a Synergy H4 Hybrid Reader (Biotek, Winooski, VT, U.S.A.). After reading, 100 μl of 0.5 N KOH was added per well to lyse the cells for protein quantification by BCA. NO production was calculated from the relative fluorescence units (RFU) and corrected for the protein content (μg). NOS-dependent NO production was calculated as the fluorescence fraction inhibited by L-NAME and presented as the fold change relative to the mean fluorescence of the non-treated cells within the same experiment.

### eNOS expression in HUVECs by in-cell Western assay

eNOS is the main enzyme generating NO in endothelial cells, thus, changes in eNOS expression may contribute to changes in NO levels [47]. Since NO contribution to vascular function was reduced by NP and PE STBEVs, the effects of STBEVs on eNOS expression in HUVECs was evaluated by In-Cell Western Assay (ICW). Confluent HUVECs at passage 3 were grown on 96-well black/clear bottom plates coated with gelatin 1%, and were cultured in PCM without ECGS and exposed to NP or PE STBEVs (100 µg/ml) or cultured without STBEVs, for 24 h. Then, media were removed, cells were rinsed with PBS (2 times) and fixed with 10% buffered formalin for 20 min. Cells were washed with tris-buffered saline (TBS, 5 min) and permeabilized with 0.1% Triton-X100. Cells were blocked with Blocking Buffer for Fluorescent Western Blotting for 1.5 h at room temperature, and then incubated overnight in agitation at 4°C with a monoclonal antibody mouse anti-eNOS (1:100 dilution, BD Biosciences, #610297) diluted in TBS-Tween (TBS-T). Next, cells were incubated with secondary antibodies (1:300, Donkey anti-Mouse IgG IRDye 800CW, LI-COR #926-32212, Lincoln, NE, U.S.A.) and CellTag 700 Stain for In-Cell Western Assay (1:800, LI-COR #926-41090) for total protein staining diluted in 25% Blocking Buffer in TBS-T. Images were taken in an LI-COR Odyssey Imaging System (LI-COR Biosciences). Mean fluorescence intensities were determined using the Image Studio Lite version 5.2 software (LI-COR Biosciences). The relative eNOS expression was calculated from the eNOS mean fluorescence/total protein fluorescence ratios and was presented as the fold change compared to the mean fluorescence of the non-treated cells within the same experiment.

### Statistical analysis

Data were analyzed using Graphpad Prism 9.3.1 (Graphpad Software, San Diego, U.S.A.). A two-way ANOVA with Sidak post-hoc test was used to assess the effect of NP versus PE STBEVs at increasing concentrations of STBEVs on vascular function (on the summary data of the *E*_max_, AUC, or EC_50_ of the MCh CCRCs). A one-way ANOVA with Dunnet post-hoc test was used to assess the effect of NP or PE STBEVs at increasing concentrations of STBEVs versus untreated vessels on vascular function, and with Sidak post-hoc test in the experiments showing a single dose of STBEVs to compare the effects of NP versus PE STBEVs, or NP and PE STBEVs versus untreated vessels. In the *in vitro* experiments, a one-way repeated measures ANOVA with Sidak post-hoc test was used to compare the effects of NP versus PE STBEVs, or NP and PE STBEVs versus untreated cells. All statistical differences were considered significant if *P*<0.05.

## Results

### Characterization of STBEVs

**Figure 1:**
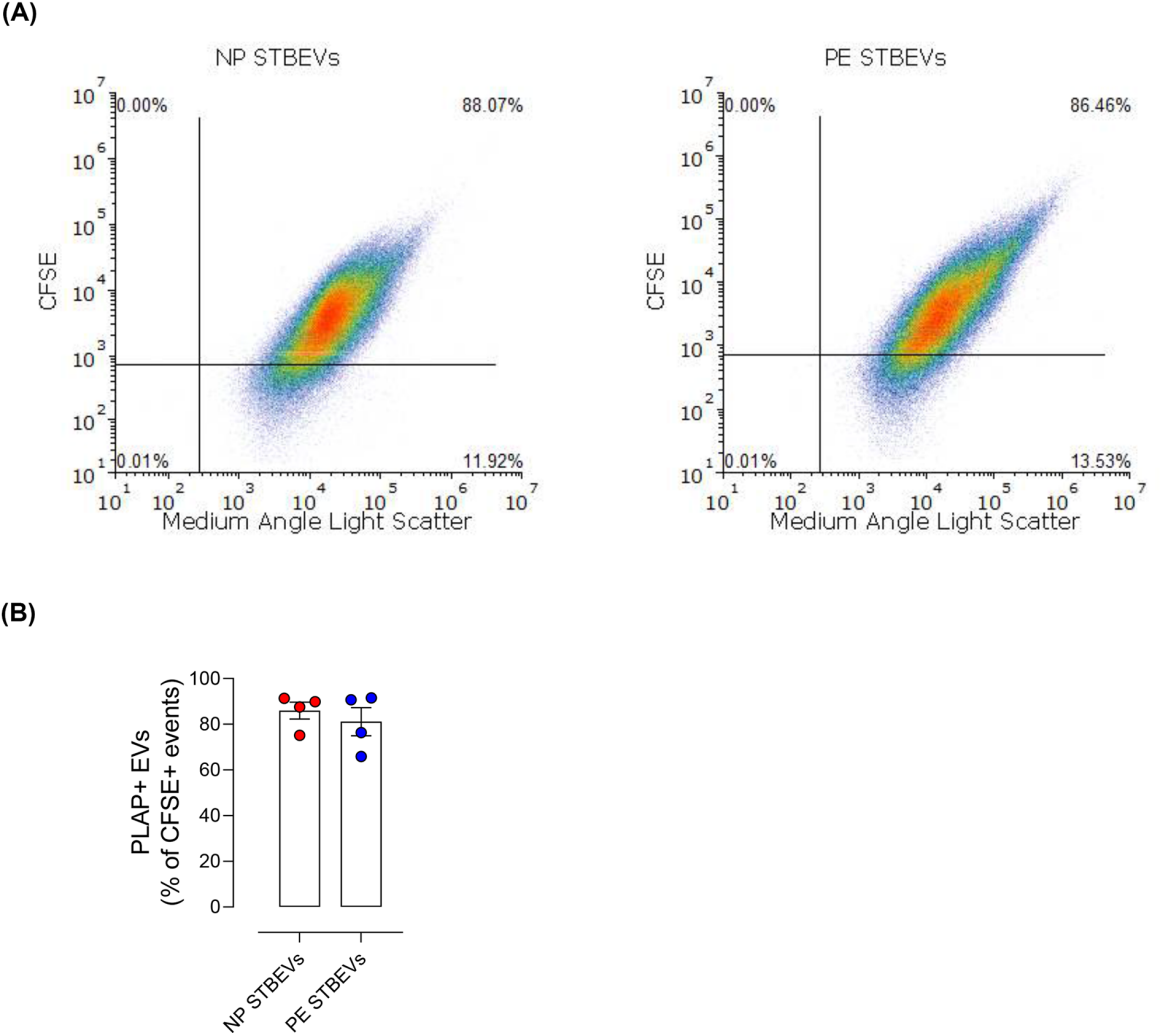
Characterization of STBEVs by microflow cytometry (**A**) Representative flow cytometry analyses showing the percentage of PLAP+ particles within the CFSE+ population (CFSE+ gated by PLAP+ particles). CFSE versus medium-angle light scatter of the STBEVs preparations (fractionated maternal side placental perfusate of samples NP3 and PE2). (**B**) Summary of percentage of PLAP positive particles among all EVs (CFSE gated by PLAP, right graph) for all NP and PE STBEVs samples.

The presence of STBEVs in our EV isolations was confirmed by microflow cytometry, NTA and by measuring the expression of EV-specific markers. For microflow cytometry, EVs were stained with CFSE, which is expected to mostly stains EVs but not protein aggregates or other membranes [40]. Microflow cytometry analysis showed that the percentage of PLAP+ particles within the CFSE+ population was 65.9–91.6% (Supplementary Figure S1A), with no differences between NP STBEVs and PE STBEVs (Figure 1A,B). The percentage of CFSE+ particles (i.e. EVs) in each of our samples was between 42.1 and 65.0%, PLAP-positive 21.8–47.5%, and double-positive (PLAP+ CFSE+) 31.6–47.1% (Supplementary Figure S1B). There were no differences in the percentage of PLAP and CFSE double-positive EVs between NP STBEVs and PE STBEVs (Supplementary Figure S1C).

**Figure 2:**
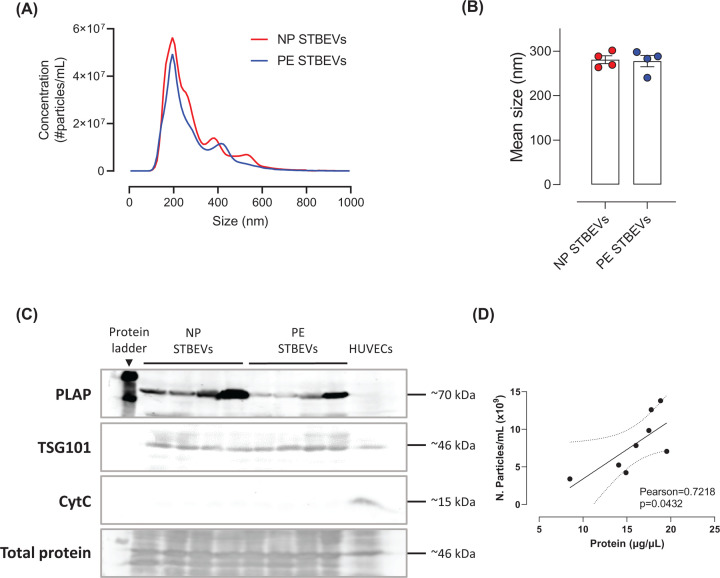
Characterization of STBEVs by NTA and Western blot (**A**) Averaged finite track length adjustment concentration/size graph for NP and PE STBEVs samples obtained by NTA. (**C**) Mean size of NP and PE STBEVs particles obtained by NTA (**B**). Western blots showing the presence or absence of PLAP, TSG101, or CytC in NP or PE STBEVs preparations from each patient. (**D**) Pearson correlation of the number of particles per ml obtained by NTA for NP and PE STBEVs preparations relative to the protein concentration obtained by the BCA assay. Dashed lines show the 95% confidence intervals. Values shown as mean ± S.E.M.

NTA analysis did not reveal any differences between NP STBEVs and PE STBEVs in the mean or modal size of the particles (Figure 2A,B and Supplementary Figure S1D). NP and PE STBEVs sizes ranged between 165.6 ± 6.9 and 164.2 ± 5.4 nm (10th percentile) to 459.2 ± 9.8 and 462.0 ± 5.4 (90th percentile), respectively; having 2–3 main populations in each sample, with peaks at 190, 380 and 530 nm for NP STBEVs, and 200 and 420 nm for PE STBEVs. In addition, all the preparations of STBEVs, as well as the HUVECs, showed to have TGS101, a marker of EVs (Figure 2C). PLAP, a marker for placental origin of the EVs, which is expressed in the syncytiotrophoblast [35], was only present in the STBEV samples but not in HUVECs (Figure 2C). CytC, which is expressed in cells but not in EVs, was not detected in the STBEVs preparations, but was expressed in HUVECs (Figure 2C). In addition, there were no differences in the total protein content versus the number of particles between NP and PE STBEVs (Supplementary Figure S1E), and there was a positive correlation between the total protein content and the number of particles in each sample (Figure 2D). Therefore, for the subsequent experiments, the concentration of STBEVs was calculated based on protein concentration of the samples.

### NP and PE STBEVs impair mesenteric artery endothelium-dependent vasodilation

**Figure 3:**
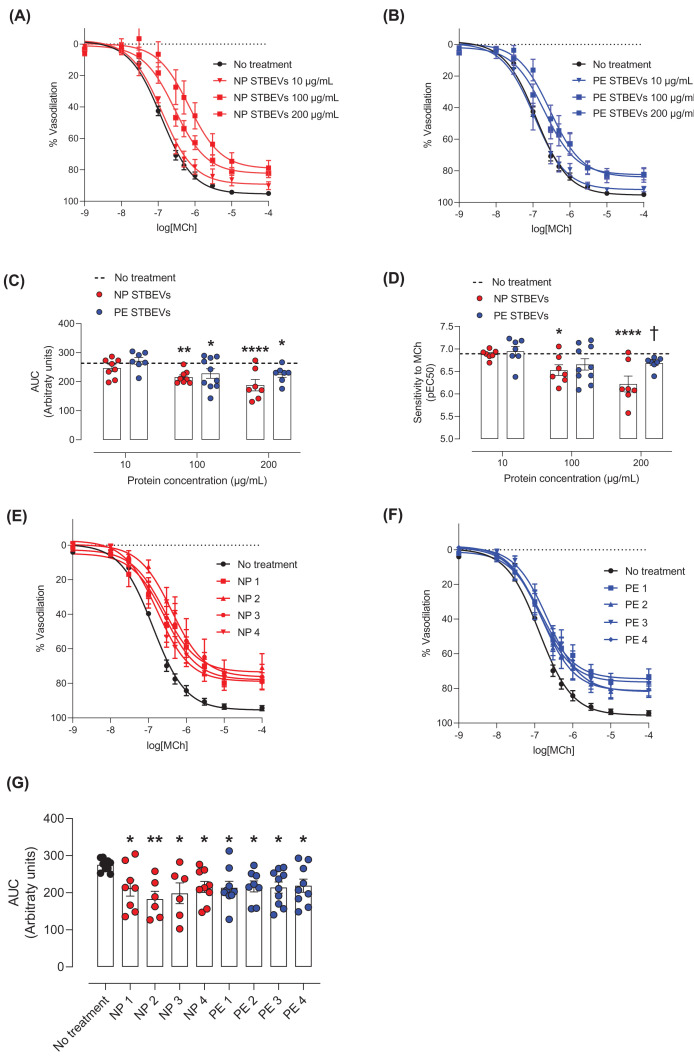
Effects of NP and PE STBEVs on vascular function in mesenteric arteries of pregnant rats (**A,B**) Cumulative concentration response curve of methylcholine (MCh)-induced vasodilation in mesenteric arteries incubated overnight with NP STBEVs (A, red) or PE STBEVs (B, blue) or without stimulus (black) at the indicated concentrations. (**C,D**) Summary graphs of the area under the curve (AUC, C) and pEC_50_ (D) obtained from concentrations curves shown in A and B. Analysis of the effect of increasing concentrations of NP or PE STBEVs compared with untreated vessels (the mean of the untreated vessels is shown as a dashed line) was done by one-way ANOVA with Dunnett post-hoc test. Comparisons of NP versus PE STBEVs at specific concentrations were analyzed by two-way ANOVA analysis with Sidak post-hoc test. *P<0.05, **P<0.01; ****P<0.0001 versus no treated vessels; †*P*<0.05 versus NP STBEVs at the same concentration. (**E,F**) Cumulative concentration response curve of MCh-induced vasodilation in mesenteric arteries incubated overnight with NP STBEVs (E) or PE STBEVs (F) from different patients. (**G**) Summary graph of AUC for all the individual samples of STBEVs. Data were analyzed by one-way ANOVA with Dunnett post-hoc test for the individual samples; *P<0.05, **P<0.01. Values shown as mean ± S.E.M.

To evaluate the effect of NP and PE STBEVs on vascular function in mesenteric arteries, a dose–response curve to increasing concentrations of STBEVs was performed (Figure 3A, B). At 100 and 200 µg/ml, both NP and PE STBEVs reduced endothelium-dependent vasodilation responses to MCh to a similar extent (Figure 3A–D and Supplementary Figure S2A,B). However, the sensitivity to MCh was reduced only by NP STBEVs (at 100 and 200 µg/mL) but not PE STBEVs (Figure 3D and Supplementary Figure S2C,D) and at 200 µg/ml, this reduction was significantly different compared with the PE STBEV-treated vessels (Figure 3D). A concentration of 100 µg/ml was chosen for the subsequent experiments since it was the lower dose where a biological impact was achieved in the vascular function assessments. The results obtained with pooled samples of NP or PE STBEVs for the dose–response curves were confirmed by assessing the effects of individual samples. The individual samples of NP and PE STBEVs showed a similar impairment in endothelium-dependent vasodilation to MCh to each other (Figure 3E–G) and to a similar extent as the pooled samples. Since the individual samples exerted a similar impairment to that induced by pooled STBEVs, pooled NP and PE STBEVs samples at 100 µg/ml were used for the subsequent experiments to reduce the numbers of biological variables.

### NP and PE STBEVs impair NO contribution to endothelium-dependent vasodilation

**Figure 4:**
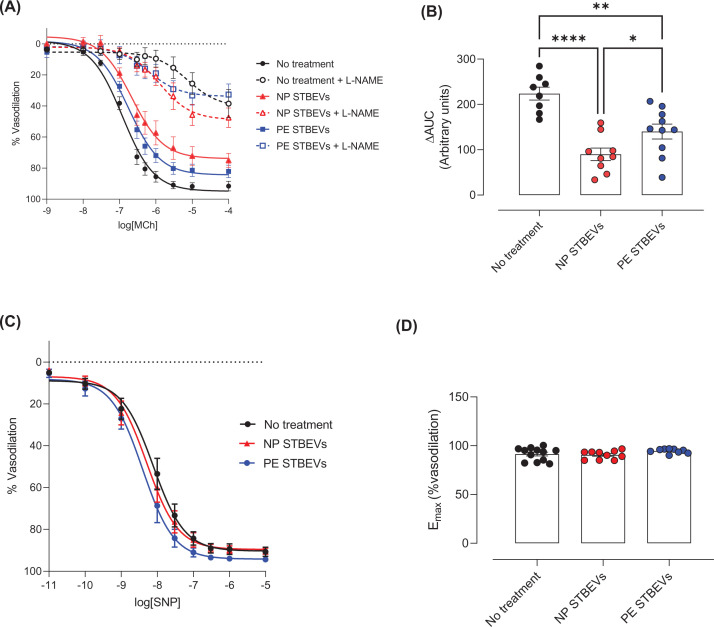
NO contribution to MCh-induced vasodilation and endothelium-independent vasodilation responses (**A**) Cumulative concentration response curve of methylcholine (MCh)-induced vasodilation in pre-constricted (with U46619) mesenteric arteries without stimulus (black) or incubated overnight with 100 µg/ml of NP STBEVs (red) or PE STBEVs (blue) and treated in the bath with (open symbols) or without (closed symbols) L-NAME (30 min before MCh-induced vasodilation). (**B**) Summary graph of nitric oxide contribution to endothelium-dependent vasodilation responses to increasing doses of MCh. Nitric oxide contribution was calculated as delta of the area under the curve (AUC) between vessels treated or not with L-NAME for each condition. Data were analyzed by one-way ANOVA with Sidak post-hoc test; *P<0.05, **P<0.01, ****P<0.0001. (**C**) Cumulative concentration response curve of sodium nitroprusside (SNP) in pre-constricted (with U46619) mesenteric arteries incubated overnight with or without 100 µg/ml of NP STBEVs (red) or PE STBEVs (blue). (**D**) Summary graph showing maximal vasodilation responses to SNP (*E*_max_) of the curves shown in (C). Values shown as mean ± S.E.M.

NO contribution to MCh-induced vasodilation was assessed by pre-treating the mesenteric arteries exposed to NP or PE STBEVs with L-NAME prior to the start of the MCh CCRC. NP and PE STBEVs both reduced NO contribution to vasodilation (i.e. the delta AUC between control and L-NAME curves), while the NO contribution after incubation with PE STBEVs was higher compared with the NP STBEVs (Figure 4A,B). Endothelium-independent vasodilation to SNP was similar between untreated and STBEV-treated mesenteric arteries (Figure 4C,D).

### NP and PE STBEVs induce endothelial dysfunction in HUVECs by activating NF-κB, increasing nitrative stress, ROS, and reducing NO bioavailability

**Figure 5:**
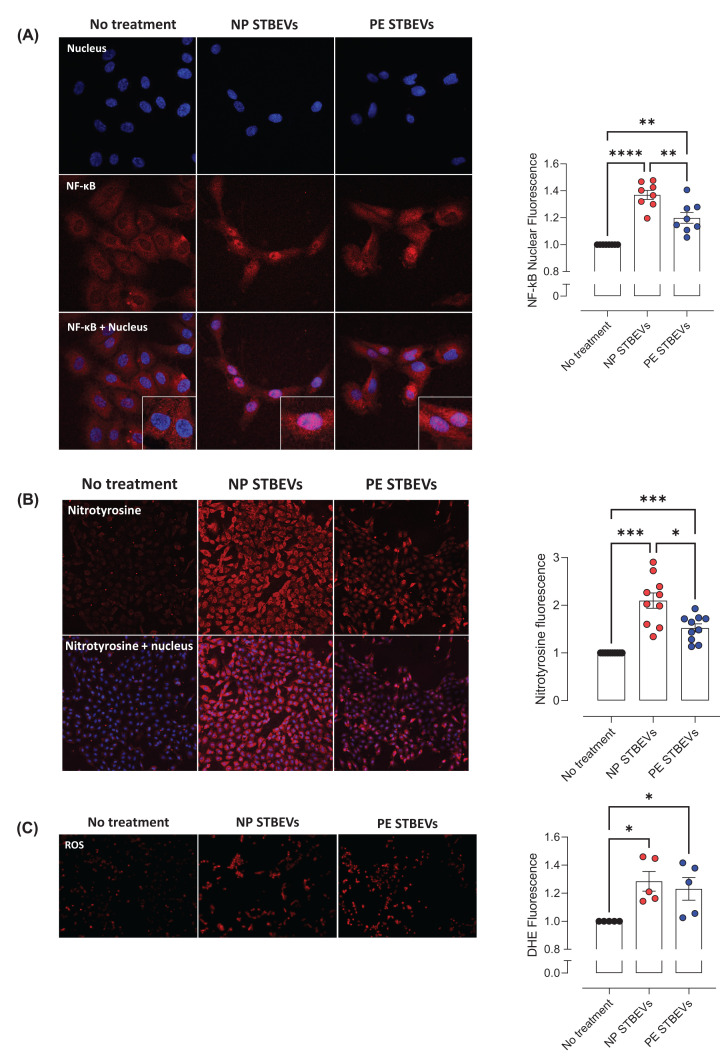
Effects of NP and PE STBEVs on NF-κB activation and ROS and nitrotyrosine levels in HUVECs (**A**) NF-κB location visualized by immunofluorescence and confocal microscopy in HUVECs treated with or without NP STBEVs or PE STBEVs (100 µg/ml) for 6 h. The graph shows the summary of the fold of changes of nuclear fluorescence relative to non-treated cells. (**B** and **C**) Immunofluorescence and confocal microscopy images for nitrotyrosine (indicative of nitrative stress, B) and DHE fluorescence (indicative of ROS levels, C) in HUVECs treated or not with NP or PE STBEVs (100 µg/ml) for 24 h. The graphs show the summary data of fold of changes of fluorescence relative to non-treated cells for nitrotyrosine (B) and DHE (C) staining. Values relative to non-treated cells and shown as mean ± S.E.M. Data were analyzed by one-way repeated measures ANOVA with Sidak post-hoc test; *P<0.05, **P<0.01, ***P<0.001, ****P<0.0001.

**Figure 6:**
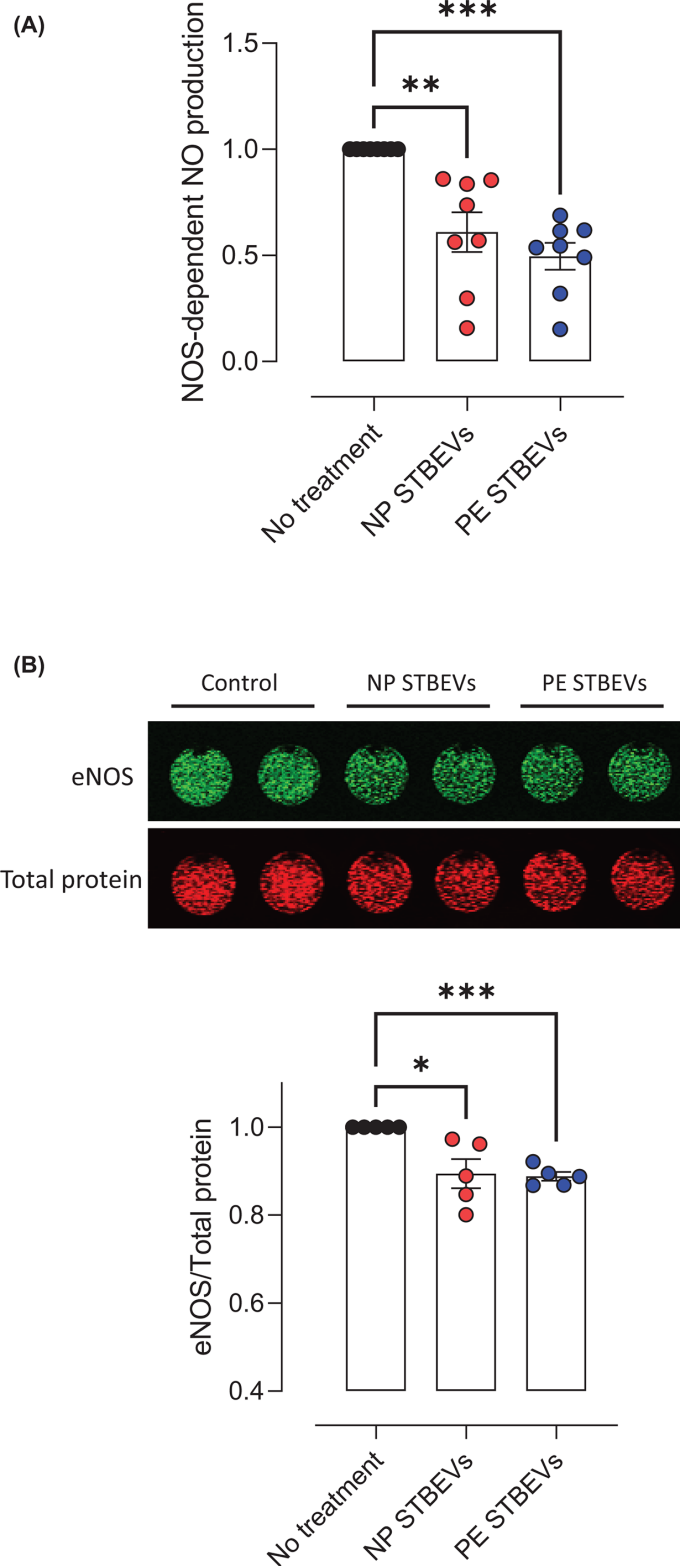
Effects of NP and PE STBEVs on NO and eNOS levels in HUVECs (**A**) Summary data of NOS-dependent NO levels in HUVECs treated or not with NP or PE STBEVs (100 µg/ml) for 24 h. (**B**) NOS-dependent NO levels were obtained from the delta between HUVECs treated and not treated with L-NAME (30 min) for each condition (data shown in Supplementary Figure S3). In-cell Western blot image for eNOS in HUVECs treated or not with NP or PE STBEVs (100 µg/ml) for 24 h. The graph shows the summary data of fold of changes of the eNOS/total protein fluorescent ratio relative to non-treated cells. Values relative to non-treated cells and shown as mean ± S.E.M. Data were analyzed by one-way repeated measures ANOVA with Sidak post-hoc test; *P<0.05, **P<0.01, ***P<0.001.

We further examined the potential mechanisms for the effects of STBEVs on endothelial function by assessing molecular markers of endothelial dysfunction in HUVECs *in vitro*. Both NP and PE STBEVs increased nuclear fluorescence for NF-κB (indicating NF-κB activation), a key signaling factor in the inflammatory response. Moreover, NF-κB activation was lower after incubation with PE STBEVs compared with incubation with NP STBEVs (Figure 5A). Similarly, NP and PE STBEVs increased nitrotyrosine levels, a marker of nitrative stress, while the nitrotyrosine levels after incubation with PE STBEVs were lower compared with the NP STBEVs (Figure 5B). Both NP and PE STBEVs increased ROS levels, a marker of oxidative stress, to a similar extent (Figure 5C). We also evaluated the *in vitro* effect of STBEVs on NOS-dependent NO production and eNOS expression. NOS-dependent NO production in HUVECs (i.e. the difference between total NO production in L-NAME non-treated or treated cells) was reduced by both NP and PE STBEVs compared with non-treated cells, and there was no difference in NOS-dependent NO production between the exposure to NP or PE STBEVs (Figure 6A). In addition, both NP and PE STBEVs reduced eNOS expression to a similar extend (Figure 6B).

## Discussion

In the present study, we sought to assess the effects of PE STBEVs on vascular endothelial function. The methodology of isolation of STBEVs and the characterization of the EVs contained in the preparations is crucial to link the causality of the effects on endothelial function with STBEVs. STBEVs were collected by placental perfusion, first described by Dragovic et al*.*, which mimics the release of STBEVs in an *in vivo* environment and thus, unlike other common methodologies such as mechanical or *ex vivo* placental explants, is thought to better represents STBEVs released into the maternal circulation *in vivo* [35,49]. It has been reported that STBEVs isolated by mechanical, culture explants or placenta perfusions protocols result in different characteristics and biological effects of STBEVs [36,49,50]. While STBEVs have been previously analyzed by flow cytometry [35], to the best of our knowledge, this is the first study reporting the characterization of large and small particles in STBEVs perfusates and comparing them between NP and PE STBEVs by microflow cytometry. Microflow cytometry allows to resolve particles scattering as little as 100 nm [51], making possible to characterize a broader spectrum of the EVs content than conventional flow cytometry. The microflow cytometry analysis showed that STBEVs preparations from placenta perfusion resulted in a high enrichment of STBEVs (65.9–91.6% of PLAP-positive EVs), which is similar to the PLAP-positivity reported by Dragovic et al. using conventional flow cytometry (mostly microvesicles 35). Thus, our data show that STBEVs preparations collected by placenta perfusion are also enriched in nano-STBEVs. As reported by Dragovic et al*.*, the PLAP-negative fraction of EVs may be composed by EVs derived from platelets, red blood cells, leukocytes, and endothelial cells [35]; which also represent a large part of the EVs released from the placenta into the bloodstream. In addition, we specifically used preparations of STBEVs containing both small and large EVs to better represent the broad spectrum of EVs released by syncytiotrophoblast cells.

To evaluate whether PE STBEVs contribute to systemic vascular dysfunction in PE, we assessed the effect of NP and PE STBEVs on systemic maternal resistance arteries of pregnant rats in *ex vivo* conditions. PE STBEVs impaired endothelium-dependent vasodilation in mesenteric arteries, and that the effect was dose-dependent and not greater than the impact of NP STBEVs. This impairment appeared to be due to a reduction in NO bioavailability. In addition, neither NP nor PE STBEVs impacted endothelium-independent vasodilation responses to SNP, suggesting that the impairment in vascular function was caused by a specific effect on the endothelium and not on the smooth muscle cells. Our findings are consistent with the study by Tong et al*.*, which reported that NP STBEVs (collected from placental explant cultures) infused *in vivo* in pregnant mice for 24 h impaired endothelium-dependent vasodilation in mesenteric arteries, and that this was also associated with a reduction in NO contribution to vasodilation [22]. Our results are also corroborated by other *ex vivo* studies reporting NP STBEVs impair vascular function in human maternal small subcutaneous fat arteries after acute exposure (2 h, STBEVs isolated by mechanical protocol) [20], or overnight incubation (at 4°C in PBS, STBEVs isolated by placental perfusion) in uterine arteries from pregnant rats [21]. Thus, the effects shown by our NP STBEVs reproduce what was previously published regarding the impairment of vascular function.

In contrast with our expectations and despite differences in the composition of STBEVs that may suggest a greater impact of PE STBEVs on vascular function than NP STBEVs [25–28], our results showed that the impairment induced by PE STBEVs was not greater than that of NP STBEVs, and even less detrimental in some parameters. These results are similar to those from two other studies that showed no differences in the uptake of NP compared with PE STBEVs, or in the expression of intercellular adhesion molecule 1 expression (ICAM-1, which is associated with endothelial dysfunction) in endothelial cells [52,53]. However, even though PE STBEVs did not have a greater impact on vascular function compared to NP STBEVs, our results show some differences in the underlying mechanisms. We found that only NP STBEVs but not PE STBEVs impaired MCh sensitivity, and that the NO contribution was less in NP STBEVs compared with PE STBEVs. The latter suggests that PE STBEVs may have a lower impact on those parameters, but since both NP and PE STBEVs have a similar impact on maximal endothelium-dependent vasodilation, this may suggest that PE STBEVs effect may also involve other pathways such as the endothelium-derived hyperpolarizing factor or prostaglandins [54], which may be an area for future research. The differential impact of PE STBEVs partially aligns with a recent study comparing effects of NP versus PE STBEVs on macrophages, which reported that NP STBEVs induced a proinflammatory response while PE STBEVs did not [55]. Thus, even though the impact of PE STBEVs on endothelial function was not greater than NP STBEVs, our results suggest there are some differences in the underlying mechanisms developing endothelial dysfunction between NP and PE STBEvs, which may result from the differences in their composition previously reported [25–28].

Our results showed that NP and PE STBEVs increased NF-κB activation, while a higher level of NF-κB activation was observed in cells exposed to NP STBEVs compared with PE STBEVs. NF-κB is a protein complex central for mediating inflammatory responses and involved in endothelial dysfunction [44]. When activated, NF-κB subunits p65 and p50 translocate to the nucleus, acting as a transcription factor of genes involved in inflammation and redox status, among other functions [44]. NF-κB activation can contribute to impaired endothelial function by increasing oxidative stress and inducing the expression of NADPH oxidase isoforms [32]. We previously reported that NP STBEVs did not increase ROS levels in HUVECs after 30 min of incubation [24]. Here, we expanded these findings showing that, after 24 h of incubation, both NP and PE STBEVs increase ROS levels; an increase in oxidative stress that aligns with the increase in NF-κB activation.

An increase in ROS in endothelial cells results in reduced NO bioavailability [13]. In line with the increase in ROS levels, we found that both NP and PE STBEVs reduced NO levels *in vitro* to the same extent. However, this is in contrast with the higher NO contribution to vasodilation after incubation with PE STBEVs compared to NP STBEVs *ex vivo*. This may be explained by differences in the experimental approach (*ex vivo* versus *in vitro* conditions), the type of endothelial cell (fetal vs. maternal), or the contribution of other cell types in the vascular function experiment. STBEVs also reduced eNOS expression, suggesting that the reduced NO levels and NO contribution to vasodilation may have been (partially) due to a reduction in eNOS expression. This reduction in eNOS expression may also be linked to the increase in NF-κB activation, as NF-κB activity has been reported to reduce eNOS expression in HUVECs by decreasing eNOS mRNA stability [31].

NO reacts with superoxide to produce peroxynitrite, which results in nitrative stress. Peroxynitrite nitrates proteins on tyrosine residues to produce the more stable product nitrotyrosine, which is used as a marker of nitrative stress [13]. We previously reported that NP STBEVs increased nitrotyrosine levels in HUVECs [24]. Our current results show that both NP and PE STBEVs increased nitrotyrosine levels, suggesting an increase in nitrative stress. However, the increase in nitrotyrosine levels after incubation with PE STBEVs was lower compared with NP STBEVs. Overall, we showed that both NP and PE STBEVs induce endothelial dysfunction via NF-κB activation, oxidative and nitrative stress, and reduced eNOS expression and NO bioavailability. However, the extent to which NP and PE STBEVs exert their effects to induce vascular dysfunction appears to be different.

Our hypothesis centered around differences between NP and PE STBEVs on vascular endothelial cell function and was thus our study protocol controlled for the dose of STBEVs. Based on our findings comparing the effects of 10 versus 100 µg/ml of both NP and PE STBEVs on vascular function, it was the concentration of STBEVs that had a greater role than the placental source (i.e. NP versus PE). It is known that the concentration of STBEVs in maternal plasma is higher in women with PE [16–19]. Thus, a higher release of STBEVs could be contributing to vascular endothelial cell dysfunction in PE.

While our study shows the impact of PE STBEVs on vascular function and different intracellular pathways in endothelial cells, the mechanisms by which PE STBEVs impact the endothelium remain to be investigated. One of these mechanisms could be the activation of the lectin-like oxidized low-density lipoprotein receptor-1 (LOX-1), a scavenger receptor that plays a role in the pathogenesis of various cardiovascular diseases [56]. LOX-1 activation induces endothelial dysfunction by mechanisms involving NF-κB activation, oxidative stress, and reduction in NO levels (reviewed in [57]), similar to the ones described for PE STBEVs in the present study. We have previously shown that NP STBEVs impair vascular function via LOX-1 [21] and that the vasculature of women with PE expresses more LOX-1 [58]. Thus, a higher expression of LOX-1 in arteries of women with PE may contribute to the detrimental effects of PE STBEVs on vascular function. However, further studies are required to elucidate whether the effects induced by PE STBEVs impact vascular function via LOX-1. Additionally, the arteries and HUVECs used in this study were isolated from women with normal pregnancies. Women with risk factors such as obesity and diabetes are at a high risk of developing PE [59]. It may be suggested that the vasculature of women prone to developing PE is predisposed to the detrimental effects of STBEVs. Thus, evaluating the response of the vasculature of women with PE (or with risk factors for developing PE) to PE STBEVs requires further investigation.

Although our findings align with some studies [53,55,60], there are various other studies suggesting a greater proinflammatory impact of PE STBEVs compared with NP STBEVs [29,61,62], which would suggest a more detrimental effect on endothelial function by PE STBEVs [63]. However, the differences between our study and others may be explained by differences in the criteria for diagnosing of PE. There is increasing evidence to suggest that PE should be classified as early-onset preeclampsia (EOPE, developed <34 weeks gestation) or late-onset preeclampsia (LOPE, developed >34 weeks gestation), which many experts in the field agree upon [33,34,64,65]. While EOPE is generally associated with more severe symptoms and complications than LOPE, it only accounts for approximately 10% of the PE cases [65], while LOPE represents the majority of the cases of PE, with 90% in high-income countries and 70% in medium-low income countries [65,66], and is rapidly increasing in developed countries [65]. Both LOPE and EOPE are proposed to result from a dysfunctional and stressed/senescent placenta [6]; while the impaired placental function in EOPE is thought to be associated with improper placentation and incomplete remodeling of the uteroplacental arteries, LOPE is thought to result from an early senescent placenta leading to impaired placental function [6]. Thus, differences in the pathophysiology of EOPE and LOPE may account for differences in the composition and effects of STBEVs. Based on the timing of delivery, the PE patients who participated in our study developed LOPE. To the best of our knowledge, this is the first study specifically using STBEVs from LOPE on vascular function (as most studies have not provided sufficient clinical data or do not distinguish between EOPE versus LOPE), which is the most common type of PE [65]. Interestingly, a recent study showed that, unlike NP STBEVs, PE STBEVs from LOPE did not induce a proinflammatory response in macrophages [55]. Further studies are necessary to explore the effects of PE STBEVs from EOPE on the vascular endothelial function, which was beyond the scope of the current project.

In conclusion, our results show that PE STBEVs induce vascular dysfunction in mesenteric arteries from pregnant rats but not at a greater extent than NP STBEVs. PE STBEVs induced vascular dysfunction by a mechanism that involves NF-κB activation, increased oxidative and nitrative stress, and reduced eNOS expression and NO bioavailability. Although NP STBEVs affected the same pathways addressed in the present study, the effect of NP STBEVs on NO contribution to vasodilation *ex vivo*, and NF-κB activation and nitrotyrosine levels in HUVECs, were greater than PE STBEVs. This may indicate that PE STBEVs are less detrimental to endothelial function and may induce changes in other endothelial vasodilation pathways such as endothelium-derived hyperpolarizing factor or prostaglandins; however, these interesting findings warrant further research. Moreover, the fact that plasmatic STBEV concentrations are higher in PE compared with normal pregnancies [17,19] suggests that PE STBEVs are likely playing a role in the vascular dysfunction in this syndrome. In addition, future studies addressing the effects of PE STBEVs in vessels from women with PE or susceptible to develop PE and the mechanisms that lead to vascular dysfunction are still required. Taken together, these data add to our understanding of the mechanisms leading to vascular dysfunction in PE and proposes PE STBEVs as a potential factor linking the placenta to the vascular dysfunction associated with PE. Our study thus contributes to the body of literature aiming to further elucidate the pathophysiology of PE with hopes of ultimately developing a therapeutic intervention to improve outcomes for women with PE and their children.

## Perspectives

- While the signs and adverse outcomes of PE are well described, a clear mechanism of the pathophysiology of PE is still lacking. It is thought that poor development of the placenta leads to a dysfunctional placenta, which may contribute to vascular endothelial dysfunction in the mother by releasing placental stress-related factors (such as STBEVs) into the maternal circulation. However, whether STBEVs from PE contribute to vascular dysfunction is still unknown.
- To the best of our knowledge, this is the first study showing that STBEVs from placentas of women with PE impair vascular endothelial function in systemic arteries. Both NP and PE STBEVs impaired vascular endothelial function in resistance arteries from pregnant rats. The underlying mechanisms involved reduced NO bioavailability, activation of NF-kB, increased ROS, nitrative stress, and reduced eNOS and NO levels.
- Even though the overall effect of PE STBEVs was not greater than NP STBEVs, the higher release of STBEVs in PE suggests they may contribute to maternal vascular dysfunction in this disorder, thereby contributing to the adverse outcomes of PE. The present study sheds light on the mechanisms that underlie vascular dysfunction in PE, ultimately contributing to the development of therapies to ameliorate or prevent the adverse outcomes in the mother and the fetus associated with this syndrome.

## Supplementary Material

Supplementary Figures S1-S2 and Tables S1-S2

## Data Availability

The authors confirm that the data supporting the findings of the present study are available within the article, supplementary materials, and from the corresponding authors Dr Christy-Lynn M. Cooke or Dr Sandra T. Davidge upon reasonable request.

## Abbreviations

AUC: area under the curve
BCA: bicinchoninic acid assay
BSA: bovine serum albumin
CCRC: cumulative concentration response curve
CFDA-SE: 5(6)-carboxyfluorescein diacetate succinimidyl ester
CFSE: carboxyfluorescein succinimidyl ester
CytC: cytochrome *c*
DAF-FM diacetate: 4-amino-5-methylamino-2′,7′-difluorofluorescein diacetate
DHE: dihydroethidium
ECGS: endothelial cell growth supplement
eNOS: endothelial nitric oxide synthase
EOPE: early-onset preeclampsia
EV: extracellular vesicle
FBS: fetal bovine serum
HUVEC: human umbilical vein endothelial cell
ICAM-1: intercellular adhesion molecule 1
ICW: in cell Western assay
ISSHP: International Society for the Study of Hypertension in Pregnancy
KPSS: high potassium salt solution
L-NAME: Nω-Nitro-L-arginine methyl ester hydrochloride
LOPE: late-onset preeclampsia
LOX-1: lectin-like oxidized low-density lipoprotein receptor-1
MCh: methylcholine
NF-κB: nuclear factor kappa-light-chain-enhancer of activated B cells
NO: nitric oxide
NOS: nitric oxide synthase
NP: normal pregnancy
NP STBEV: syncytiotrophoblast-derived extracellular vesicles from normal pregnancy
NTA: nanoparticle tracking analysis
PBS: phosphate-buffered saline
PCM: primary culture medium
PE: preeclampsia
PE STBEV: syncytiotrophoblast-derived extracellular vesicles from preeclampsia
PLAP: placental alkaline phosphatase
PMSF: phenylmethylsulfonyl fluoride
PSS: physiological salt solution
RFU: relative fluorescence units
ROS: reactive oxygen species
SDS: sodium dodecyl sulfate
SMC: smooth muscle cell
SNP: sodium nitroprusside
STBEV: syncytiotrophoblast-derived extracellular vesicle
TBS: Tris-buffered saline
TSG101: tumor susceptibility gene 101
