# Supplementary material for: Placenta-derived extracellular vesicles from preeclamptic and healthy pregnancies impair ex vivo vascular endothelial function
Source: Biosci Rep. 2022 Dec 16;42(12):BSR20222185. doi: 10.1042/BSR20222185 (PMC9760606; doi:10.1042/BSR20222185)
Supplement: Supplementary Figures S1-S2 and Tables S1-S2 [file BSR-2022-2185_supp.pdf]

**Supplement figures 1**

# Figure S1

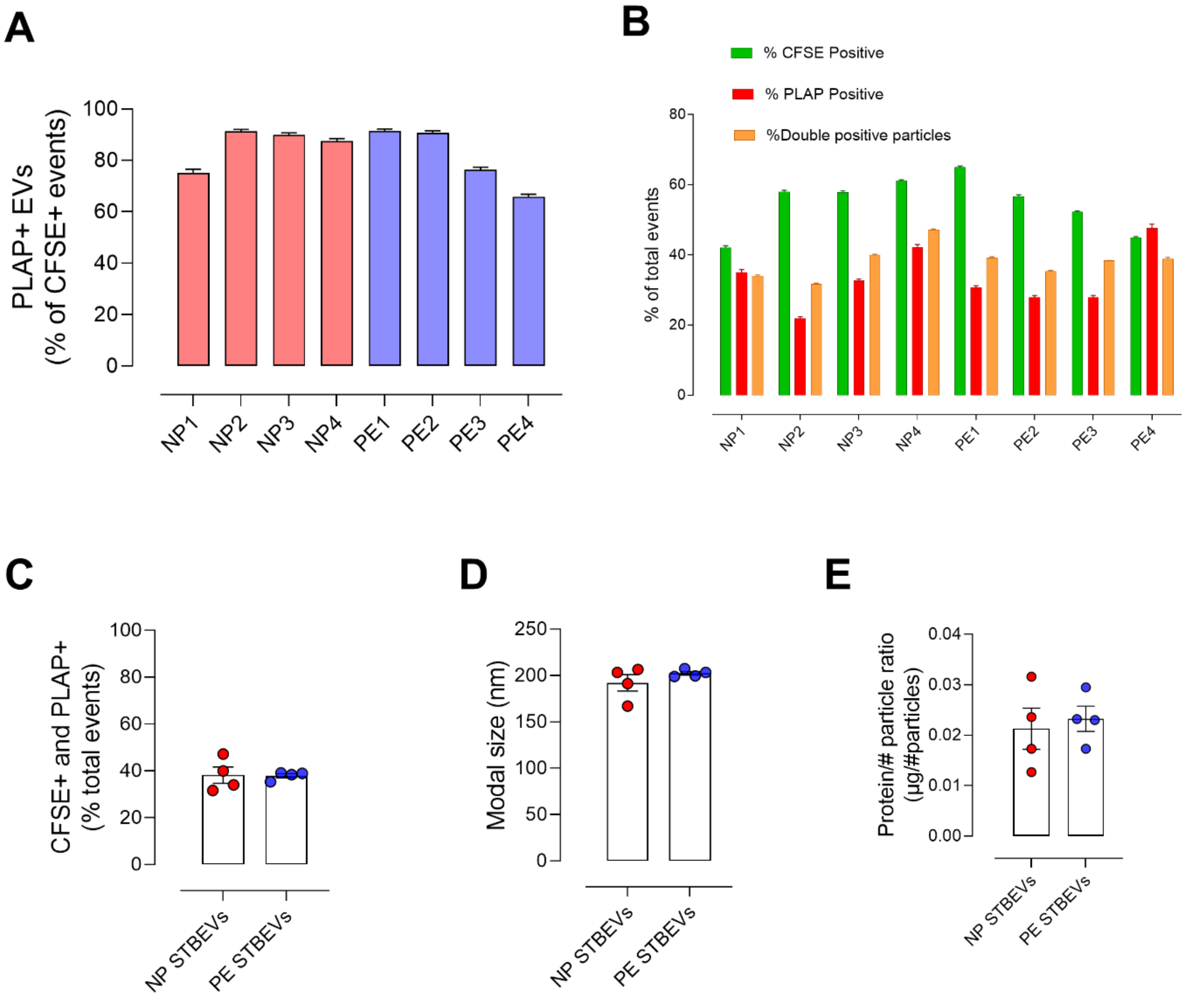

**Fig S1. Characterization of STBEVs. (A)** Summary graph of % of PLAP positive particles among all EVs (CFSE gated by PLAP) per sample. **(B)** Summary graphs showing the % of particles positive for CFSE, PLAP and double positive per sample. **(C)** Graph showing the percentage of CFSE and PLAP positive events relative to total events. **(D)** Modal size of NP and PE STBEVs obtained by NTA. **(E)** Summary of the ratio of protein concentration obtained by BCA vs. number of particles obtained by NTA. Values shown as mean±S.E.M.

# Figure S2

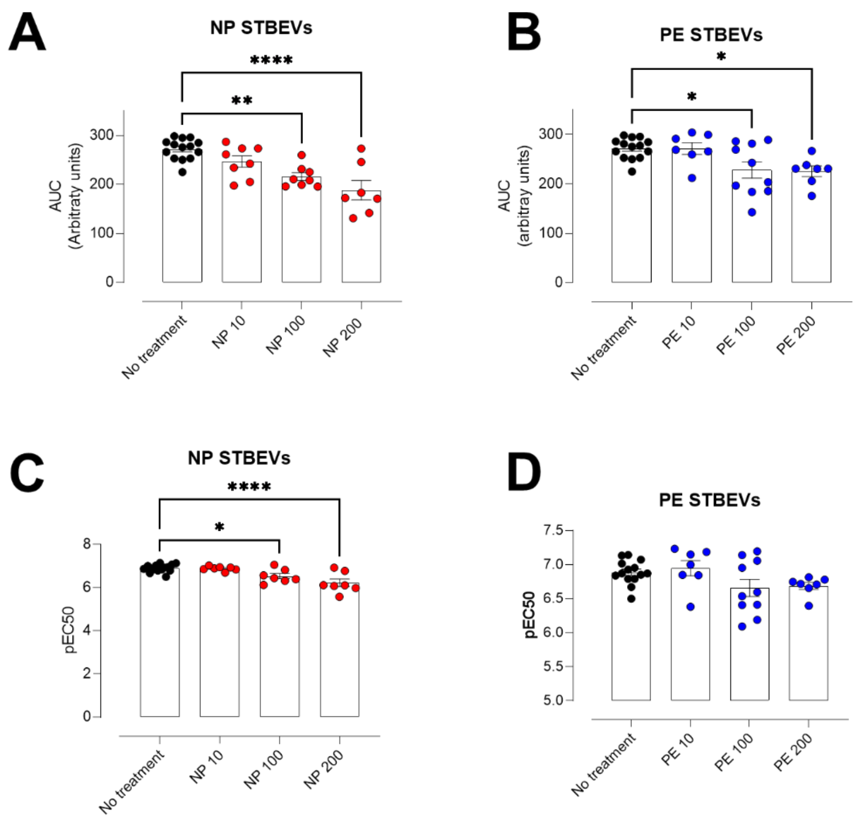
A
NP STBEVs
****
**
AUC (Arbitraty units)
300
200
100
0
No treatment
NP 10
NP 100
NP 200
B
PE STBEVs
*
*
AUC (arbitray units)
300
200
100
0
No treatment
PE 10
PE 100
PE 200
C
NP STBEVs
****
*
pEC50
8
6
4
2
0
No treatment
NP 10
NP 100
NP 200
D
PE STBEVs
pEC50
7.5
7.0
6.5
6.0
5.5
5.0
No treatment
PE 10
PE 100
PE 200

**Fig S2. Effects of NP and PE STBEVson vascular function in pregnant rat mesenteric arteries. (A-D)** Summary graphs showing the AUP (A,B) or $pEC_{50}$ (C,D) of the endothelium-dependent response to MCh curves in vessels treated or not overnight with NP STBEVs (A) or PE STBEVs (B). The values were obtained from the data showed in Fig 3A,B. Statistical analysis of the effect of increasing concentrations of NP or PE STBEVs compared to untreated vessels (mean of untreated vessels shown as a dashed line) was done by one-way ANOVA with Sidak post hoc test. Values shown as mean±S.E.M. Data of the NP or PE STBEVs vs. non-treated vessels were analyzed by one-way ANOVA with Sidak post hoc test, and NP vs PE STBEVs were done by two-way ANOVA with Sidak post hoc test. * $p<0.05$; ** $p<0.01$; ***$p<0.001$; ****$p<0.0001$.

| **Supplementary Table 1: MicroFlow Cytometer Settings** | | | | |
|---|---|---|---|---|
| **Platform** | Apogee A60 MP | S/N 00130 | | |
| | | | | |
| **Parameter** | **Setting** | | | |
| Sample Flow Rate | 3.01 µL/min | | | |
| Pressure | 150 units | | | |
| Acquisition time | 60 sec | | | |
| Sample Dilution | 100x with PBS | | | |
| Volume/well | 250 µL | | | |
| Sample volume | 10 µL | | | |
| Diluent volume | 990- Ab vol µL | | | |
| Event Trigger | LALs unless stated otherwise | | | |
| | | | | |
| **Channel** | **Laser Power (mW)** | **PMT** | **Gain** | **Threshold** |
| 405nm | 85 | | | |
| 488nm | 70 | | | |
| 561nm | 75 | | | |
| 638nm | 70 | | | |
| 405-SALS | | 360 | 1.0 | NA |
| 405-LALS | | 375 | 1.0 | NA |
| 405-MALS | | 380 | 1.0 | 29 |
| 405-Blue | | 505 | 1.0 | |
| 405-Green | | 575 | 1.0 | |
| 488-Green | | 480 | 1.0 | |
| 488-Orange | | 300 | 1.0 | |
| 488-Red | | 310 | 1.0 | |
| 561-Orange | | 510 | 1.0 | |
| 561-Red | | 770 | 1.0 | |
| 638-Red | | 500 | 1.0 | |
| 638-Far Red | | 300 | 1.0 | |
| | | | | |
| **Beads** | **Product number** | **Lot number** | **Expiration** | |
| Monitoring | Apogee 1527 | CAL0145 | 27 May 2025 | |
| Calibration | Apogee 1524 | CAL0143 | 15 May 2025 | |
| Calibration | Spherotech RCP05 | AL01 | 01 June 2023 | |

| Supplementary Table 3: MIFlowCyt / MISEV Compliant Items | |
|---|---|
| **Requirement** | **Please Include Requested Information** |
| 1.1. Purpose | To confirm and characterize the content of syncytiotrophoblast extracellular vesicles (STBEVs) in placental perfusates from pregnant women with normal pregnancies or preeclampsia. |
| 1.2. Keywords | Syncytiotrophoblast extracellular vesicles, placenta, preeclampsia. |
| 1.3. Experiment variables | Placental alkaline phosphatase (PLAP) and Carboxyfluorescein succinimidyl ester (CFSE) staining. |
| 1.4. Organization name and address | Davidge Laboratory, Division of Reproductive Sciences, Department of Obstetrics & Gynecology, 232 Heritage Medical Research Centre (HMRC), University of Alberta. |
| 1.5. Primary contact name and email address | Sandra Davidge, sdavidge@ualberta.ca |
| 1.6. Date or time period of experiment | 2018 - 2022 |
| 1.7. Conclusions | Preparations from placental perfusate contained a percentage of double-positive (PLAP+ CFSE+) particles between 31.6-47.1% 42.1-65.0%. The percentage of PLAP+ particles within the CFSE+ population (STBEVs) was 65.9-91.6%. |
| 1.8. Quality control measures | Apogee Bead (1524,1527), MESF beads (Spherotech RCP-05), daily instrument monitoring for cleanliness and diluent cleanliness. Reagent and assay controls were also executed. |
| 2.1.1.1. (2.1.2.1., 2.1.3.1.) Sample description | |
| 2.1.1.2. Biological sample source description | Human derived syncytiotrophoblast derived Extracellular Vesicles (STBEV) |
| 2.1.1.3. Biological sample source organism description | Human |
| 2.1.2.2. Environmental sample location | NA |
| 2.3. Sample treatment description | <u>Human STBEVs samples</u><br>1. Frozen STBEVs samples were thawed, incubated with mouse anti-human PLAP (11.9ug/mL for 20 minutes, and then 4.55ug/mL Goat anti-mouse secondary-APC followed by CFSE (6.25uM) for 30 minutes at 37°C.<br>2. After probe(s) incubation, samples were diluted 100-fold in double-filtered (0.1 µm) phosphate-buffered saline (PBS) and analyzed with the Apogee A60 microflow cytometer using a flow rate of 3.01 µL/minute. Samples were run for at least 60 seconds. |

| | |
|---|---|
| | 3. Samples were assayed in triplicate. Conventional manual gating analysis of FCM data was performed using Histogram version 6.049 software (Apogee Flow Systems). |
| 2.4. Fluorescence reagent(s) description | 1. Spherotech RCP-05, Lot AL01<br>2. Goat anti-mouse secondary antibodies (APC Product #405308 Biolegends), AF647 (A21235 ThermoFisher), AF546 (A11030 ThermoFisher)<br>3. CFDA-SE ThermoFisher (V12883, ThermoFisher, membrane stain. |
| 3.1. Instrument manufacturers, model | 1. Apogee A60 MicroPlus Microflow cytometer (S/N 0130): Apogee Flow Systems |
| 3.3. Instrument configuration and settings | See Tables |
| 4.1. List-mode data files | |
| 4.2. Compensation description | No compensation |
| 4.3. Data transformation details | Data were analysed using Apogee Flow Systems Histogram version 6.049 software, Becton Dickinson & Company (BD) FlowJo version (build) 10.8.1, De Novo Software FCS Express RUO edition version 7.14.0020, GraphPad Prism version 9.4.0(673) for Windows. |
| 4.4.1. Gate description | Defined by unstained controls, reagent only controls. Quadrants express percent of total parent population. |
| 4.4.2. Gate statistics | Data provided as concentration (events/uL) or MESF equivalents |
| 4.4.3. Gate boundaries | Unstained controls or reagent only controls |

**Supplementary Information for Instrument Set-Up and Optimization**

(S1) Instrument Suitability for Small Particle Measurements

An Apogee A60 MicroPlus flow cytometer with 3 light scatter collect angles was used to collect small particle flow cytometry data. The Apogee bead mix (Product 1527) consists of a range of polystyrene beads (83nm, 110nm, 500nm (Figure S1, Panel 2 Gates 1, 8,9) and a range of silica beads (180nm, 240nm, 300nm, 590nm, 880, 1300nm (Figure S1, Panel 2 Gates 2-7). For sizing estimates of particles by flow cytometry, detected events were in the approximate scatter intensity range of 83nm polystyrene and 500nm polystyrene (FigureS1 A,B). Platform cleanliness was maintained using a daily cleaning protocol in addition to the on-board cleaning steps associated with start up and shut down. Prior to any sample assays, 1% bleach was used to clean any overnight biofilm build-up that may have occurred. Following bleach, multiple runs of PBS and sheath (100nm filtered $H_2O$/sodium azide (or ProClin300)). Instrument cleanliness was gauged by the event rate of the sheath (acceptance criteria <400events/second) (Figure S1C). Diluent cleanliness (acceptance criteria <400events/second) was similarly assessed (Figure S1D). The platform was also calibrated each day to provide a consistent scatter intensity for fluorescent and non-fluorescent beads. If needed, PMT voltages were automatically adjusted to achieve the desired pre-defined decade placement. During the calibration, bead fluorescent intensities and non-fluorescent intensities were compared with population counts to provide an additional measure of instrument cleanliness. Instrument stability during the time period of the analyses was demonstrated as MFI variability for 110nm (488-Green) PS bead and 2000nm (638-Red) PS was measured at <10% (Figure S1E).

(S2) Fluorescence Calibration

Fluorescence sensitivity was calibrated using Spherotech reference beads (RCP-05, LotAL01) and the related data presented in Figure S2. RCP-05 beads do not have distinct QC validated MEF values but rather utilize a cross calibration estimation using URQP particles (URQP-38-6K) (personal communication with M. Collism Spherotech Application Specialist). The log transformations of the specified molecules of equivalent soluble fluorophore (ERF) values versus log transformed of the associated median fluorescent intensities (MFI) for FITC (Figure S2A) and APC (Figure S2B); The MESF values for phycoerythrin (PE) and AlexaFluor (AF)546 were similarly derived (graphs not shown). All transformations had goodness of fit values >0.99. The slope of the line and intercept values are used to transform FL intensity data to MESF equivalents using the illustrated formula where $Int_{\mathrm{MESF}}$= calibrated intensities, a is the slope of the regression line, b is the intersect and $\mathrm{Int}_{\mathrm{measured}}$ are the measured fluorescent intensities.

$$Int_{MESF} = 10^{a \cdot log_{10}\,(Int_{measured})+b}$$

Representative samples of normal and preeclampsia derived Syncytiotrophoblast derived Extracellular Vesicles (STBEVs) is provided to illustrate the uncalibrated (C,D) and calibrated data transformations (E,F). Note that the relative percentages in each quadrant do not change with MESF transformations.

(S3) Reagent titrations

All reagents were titrated to determine the optimal concentration. CFDA-SE (5(6)-carboxyfluorescein diacetate succinimidyl ester), a stable, cell-permeable diacetate precursor to CFSE was titrated to define the optimal concentration for staining. Upon diffusion into the cell, intracellular esterases cleave the acetate group to generate CFSE, which interacts with cellular amines via its succinimidyl groups to generate a highly fluorescent green dye that is impermeant to the cell membrane. Samples (20uL) were mixed with serially diluted CFDA-SE dye (10uL) and then incubated at 37°C for 30 minutes. Samples were then diluted with PBS and assayed by flow cytometry. Titration data for CFSE concentration ((reaction final concentration before dilution, µM x MFI (Figure S3A), x separation index (Figure S3B), x counts (Figure S3C)) is presented. Similarly, samples (20uL) were mixed with serially diluted PLAP primary antibody (NDOG2, Sunderland et al., 1984, Dragovic et al., 2015) (1uL) and then incubated at ambient temperature (~18-25°C) for 20 minutes. Samples were then incubated with Goat anti Mouse secondary antibody (APC Product #405308 Biolegend) at ambient temperature (~18-25°C) for 20 minutes and then diluted with PBS and assayed by flow cytometry. Titration data for PLAP primary antibody concentration ((reaction final concentration before dilution, µM x MFI (Figure S3D), x separation index (Figure S3E), x counts (Figure S3F)) are presented. Finally, samples (20uL) were mixed with optimally diluted PLAP primary antibody (NDOG2, Sunderland et al., 1984, Dragovic et al., 2015) (1uL) and then incubated at ambient temperature (~18-25°C) for 20 minutes. Samples were then incubated with serially diluted Goat anti Mouse secondary antibody (APC Product #405308 Biolegend) at ambient temperature (~18-25°C) for 20 minutes and then diluted with PBS and assayed by flow cytometry. Titration data for GαM (-APC) secondary antibody concentration ((reaction final concentration before dilution, µg/mL x MFI (Figure S3G), x separation index (Figure S3H), x counts (Figure S3I)) are presented.

Supplementary Figure S4 Assay Controls

Specific assay controls were executed. Data are presented to illustrate the minimal to non-influence of sample diluent (PBS, Figure S4A), CFSE stain alone in the diluent (Figure S4B), secondary antibody alone (Figure S4C), or PLAP primary antibody alone (Figure S4D). Data are presented using arbitrary intensity units for relevant filter sets. PLAP primary antibody gave similar data for the 638nm (-APC) filter set as shown for the 488nm (-CFSE) filter set. Isotype controls were not included (REF is Cytometry B Clin Cytom. 2009 Nov;76(6):355-64. doi: 10.1002/cyto.b.20485).

Supplementary Figure S5 Additional Assay Controls

Representative unstained patient samples were first analyzed by light scatter (Medium Angle Light Scatter (MALS, SSC) x Small Angle Light Scatter (SALS (FSC)) (Figure S5A) and in the relevant fluorescent channels PLAP+ (APC) and CFSE+ (Figure S5B). Representative data illustrating samples stained with CFSE membrane stain only are shown in Figure S5C; approximately 80% of events are positive for the membrane stain and no positive PLAP staining as expected. Likewise,

staining STBEVs with CFSE and only the primary antibody yields only CFSE positive data but no PLAP positive data (Figure S5D). Inclusion of the secondary antibody results in a significant PLAP+ (APC) population and CFSE+, APC+ population (Figure S5E). Finally, addition of TritonX100 (2%) to the reaction mix completely changes the positivity profile for both CFSE+ and APC+ populations (FigureS5F) demonstrating that positive events were indeed derived from membrane bound particles.

Supplementary Figure S6 Additional Assay Controls (secondary antibody)

The use of different secondary antibodies for detection of PLAP+ extracellular vesicles was analyzed. Representative samples treated with CFSE+ alone are presented with the relevant PLAP+ analysis filter: AlexaFluor (AF) 647 using the APC filter set (Figure S6A) and AF546 using the TRITC filter set (FigureS6B). Complete reactions with primary, secondary and CFSE dye are shown for both secondaries are shown (Figures S6C, and S6D). Both reactions show >70% CFSE positivity and >30% positivity for PLAP. Double positivity was >30% for both. The addition of TritonX100 detergent completely changed the positivity profile for both reactions illustrating that the positive events were likely due to membrane bound vesicles.

**Figure S1**

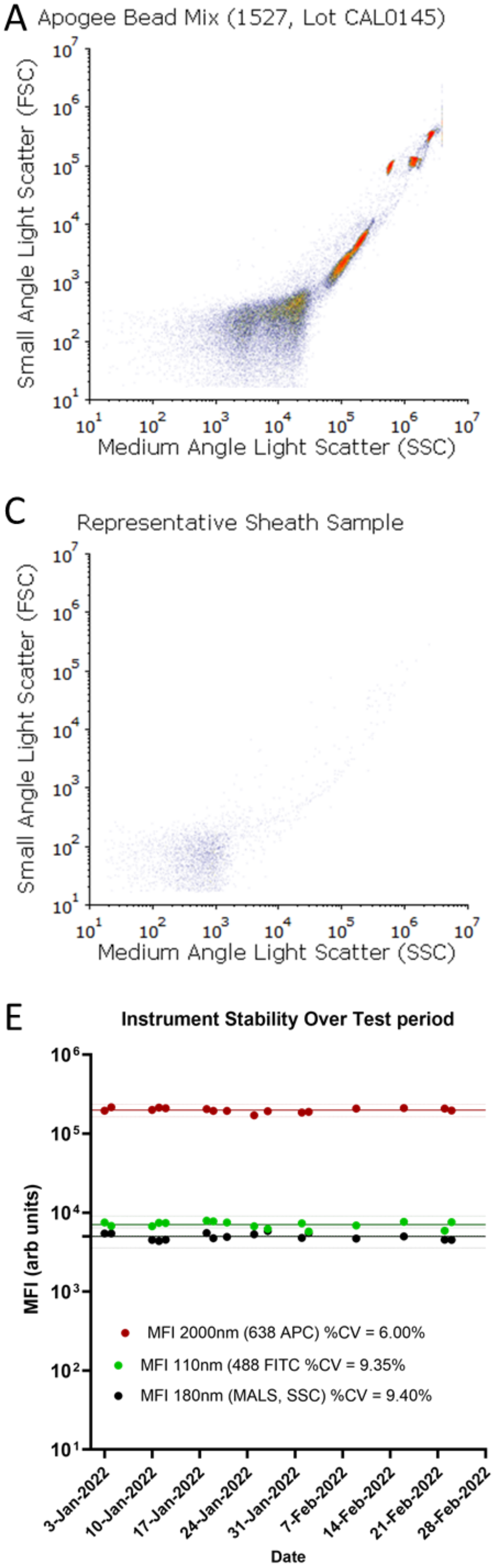

A
Apogee Bead Mix (1527, Lot CAL0145)
Small Angle Light Scatter (FSC)
Medium Angle Light Scatter (SSC)
B
Apogee Bead Mix (1527, Lot CAL0145)
488nm Green Intensity (Arb units)
Medium Angle Light Scatter (SSC)
1
2
3
4
5
6
7
8
9
C
Representative Sheath Sample
Small Angle Light Scatter (FSC)
Medium Angle Light Scatter (SSC)
D
PBS Diluent Alone
Small Angle Light Scatter (FSC)
Medium Angle Light Scatter (SSC)
E
Instrument Stability Over Test period
MFI (arb units)
MFI 2000nm (638 APC) %CV = 6.00%
MFI 110nm (488 FITC %CV = 9.35%
MFI 180nm (MALS, SSC) %CV = 9.40%
3-Jan-2022
10-Jan-2022
17-Jan-2022
24-Jan-2022
31-Jan-2022
7-Feb-2022
14-Feb-2022
21-Feb-2022
28-Feb-2022
Date

**Figure S2**

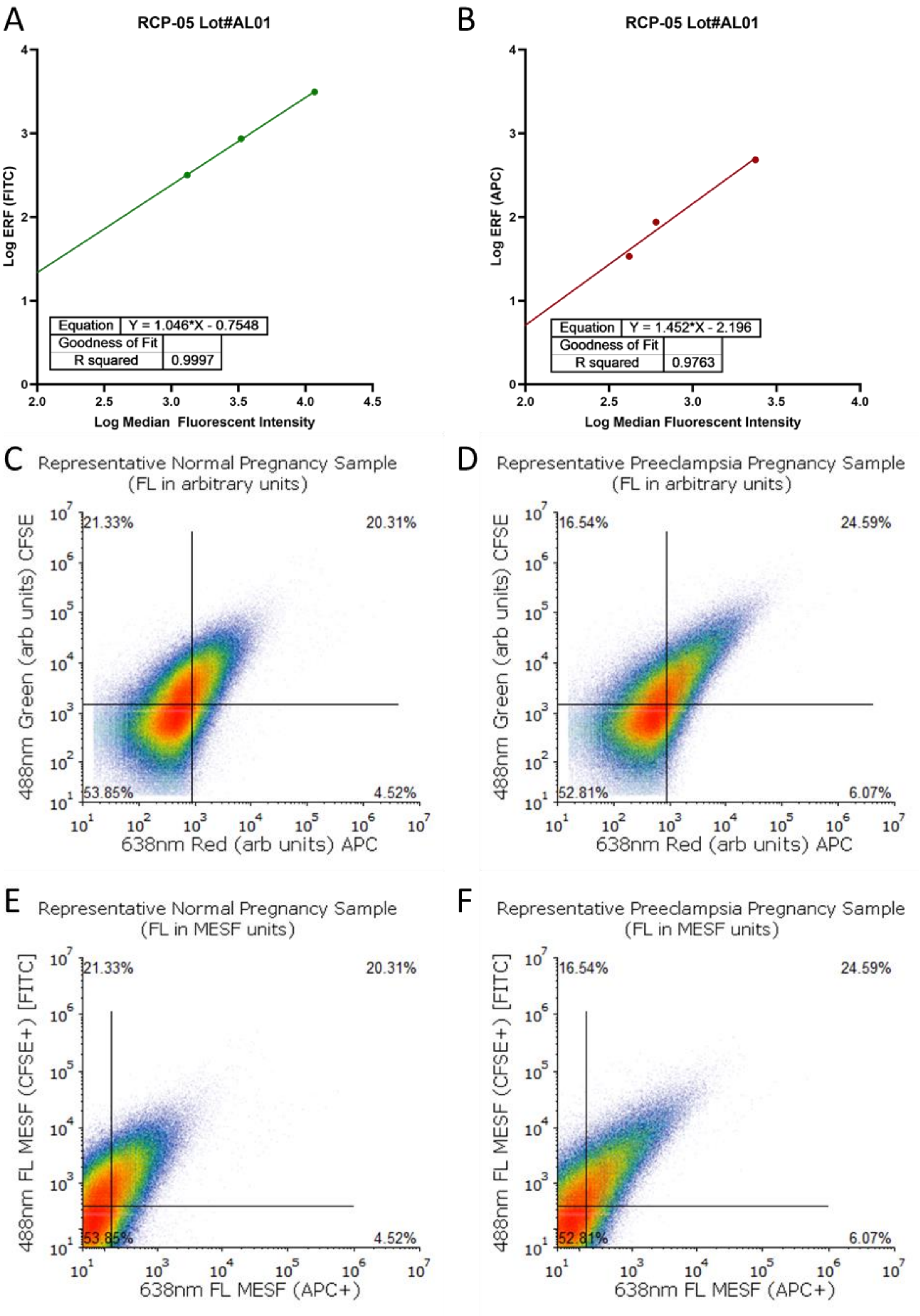
A
RCP-05 Lot#AL01
Log ERF (FITC)
Log Median Fluorescent Intensity
Equation | Y = 1.046*X - 0.7548
Goodness of Fit
R squared | 0.9997
B
RCP-05 Lot#AL01
Log ERF (APC)
Log Median Fluorescent Intensity
Equation | Y = 1.452*X - 2.196
Goodness of Fit
R squared | 0.9763
C
Representative Normal Pregnancy Sample
(FL in arbitrary units)
488nm Green (arb units) CFSE
638nm Red (arb units) APC
21.33%
20.31%
53.85%
4.52%
D
Representative Preeclampsia Pregnancy Sample
(FL in arbitrary units)
488nm Green (arb units) CFSE
638nm Red (arb units) APC
16.54%
24.59%
52.81%
6.07%
E
Representative Normal Pregnancy Sample
(FL in MESF units)
488nm FL MESF (CFSE+) [FITC]
638nm FL MESF (APC+)
21.33%
20.31%
53.85%
4.52%
F
Representative Preeclampsia Pregnancy Sample
(FL in MESF units)
488nm FL MESF (CFSE+) [FITC]
638nm FL MESF (APC+)
16.54%
24.59%
52.81%
6.07%

**Figure S3**

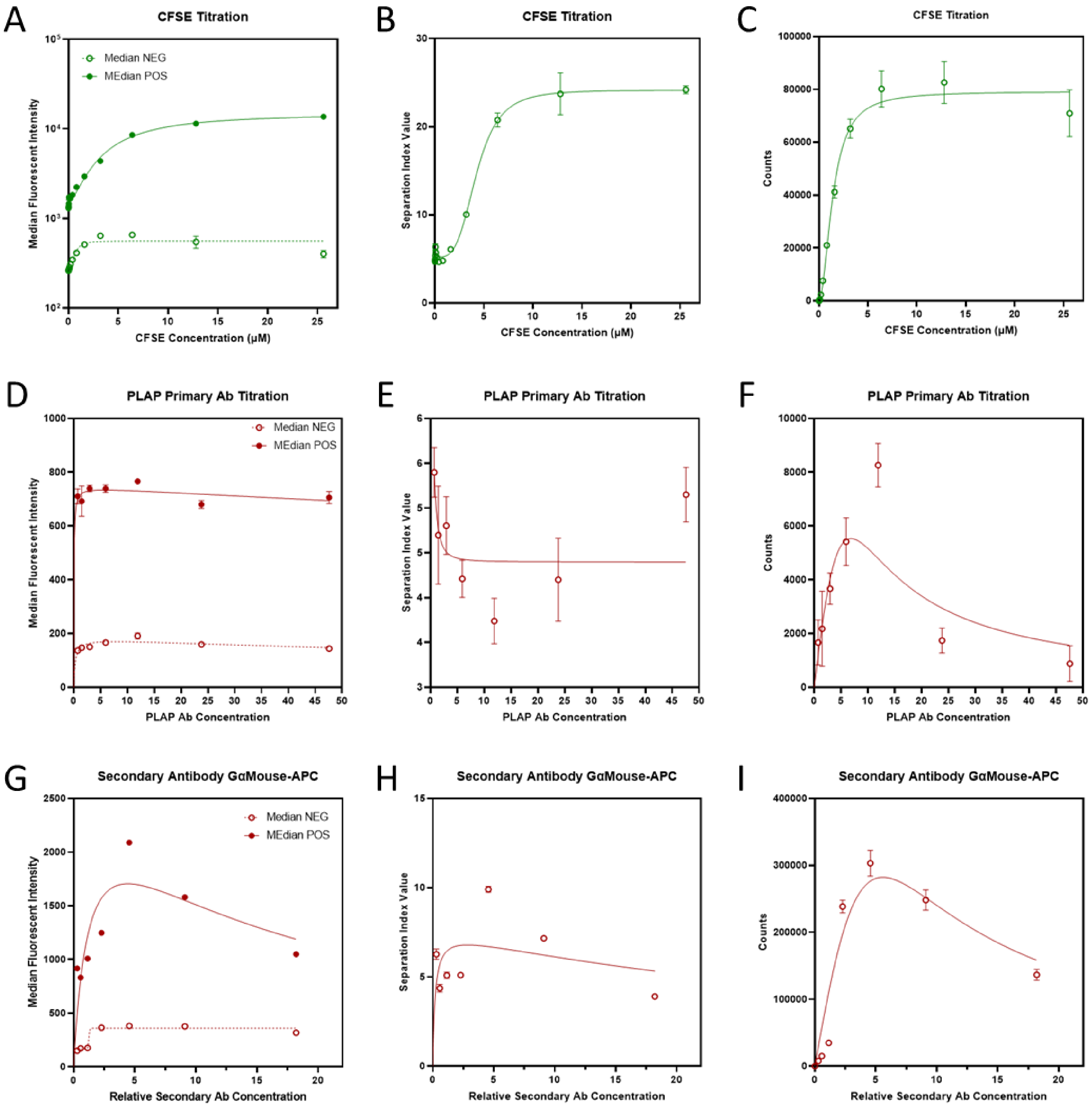
A
CFSE Titration
Median NEG
MEdian POS
Median Fluorescent Intensity
CFSE Concentration (μM)
B
CFSE Titration
Separation Index Value
CFSE Concentration (μM)
C
CFSE Titration
Counts
CFSE Concentration (μM)
D
PLAP Primary Ab Titration
Median NEG
MEdian POS
Median Fluorescent Intensity
PLAP Ab Concentration
E
PLAP Primary Ab Titration
Separation Index Value
PLAP Ab Concentration
F
PLAP Primary Ab Titration
Counts
PLAP Ab Concentration
G
Secondary Antibody GαMouse-APC
Median NEG
MEdian POS
Median Fluorescent Intensity
Relative Secondary Ab Concentration
H
Secondary Antibody GαMouse-APC
Separation Index Value
Relative Secondary Ab Concentration
I
Secondary Antibody GαMouse-APC
Counts
Relative Secondary Ab Concentration

**Figure S4**

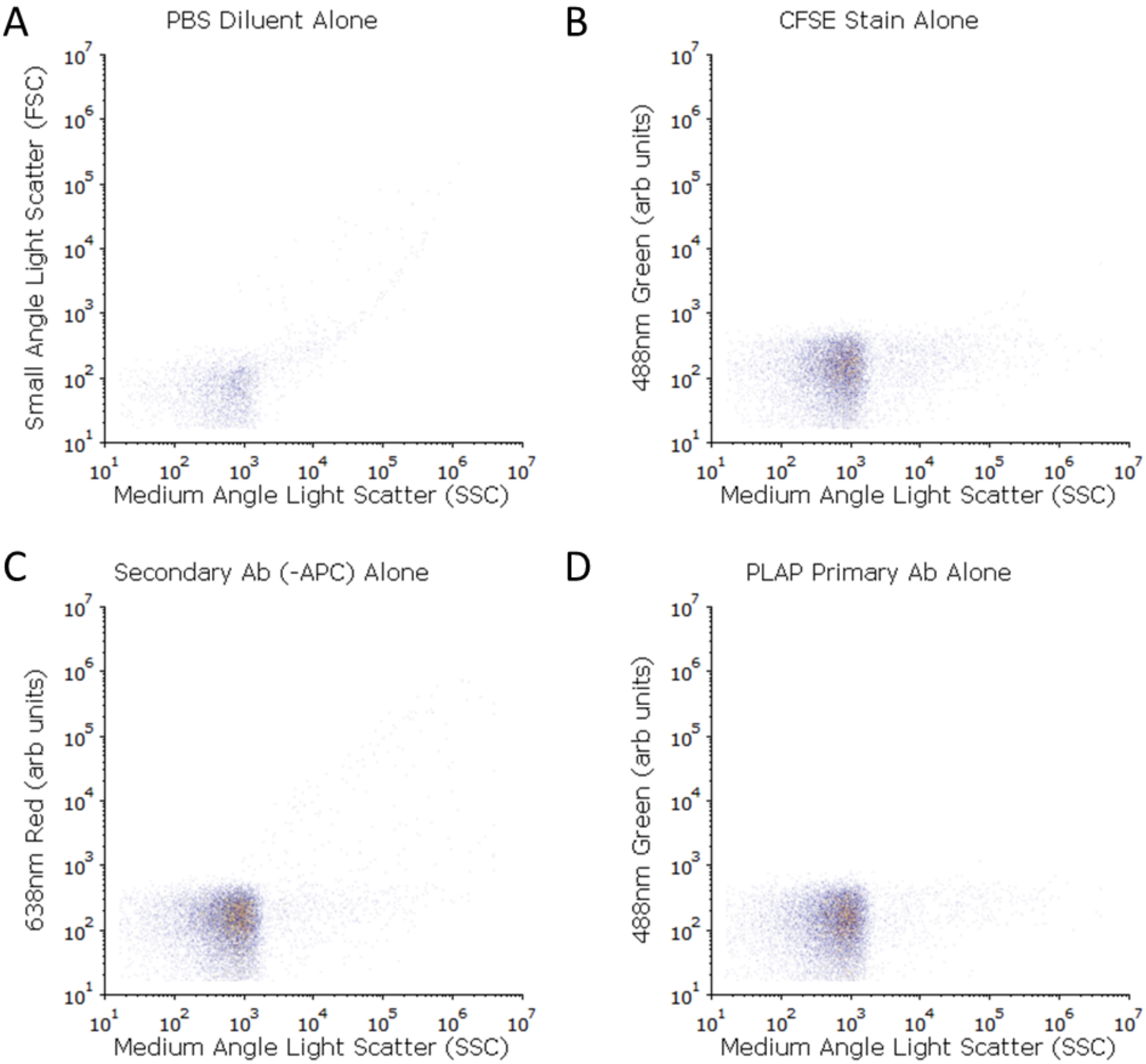
A
PBS Diluent Alone
Small Angle Light Scatter (FSC)
Medium Angle Light Scatter (SSC)
B
CFSE Stain Alone
488nm Green (arb units)
Medium Angle Light Scatter (SSC)
C
Secondary Ab (-APC) Alone
638nm Red (arb units)
Medium Angle Light Scatter (SSC)
D
PLAP Primary Ab Alone
488nm Green (arb units)
Medium Angle Light Scatter (SSC)

**Figure S5**

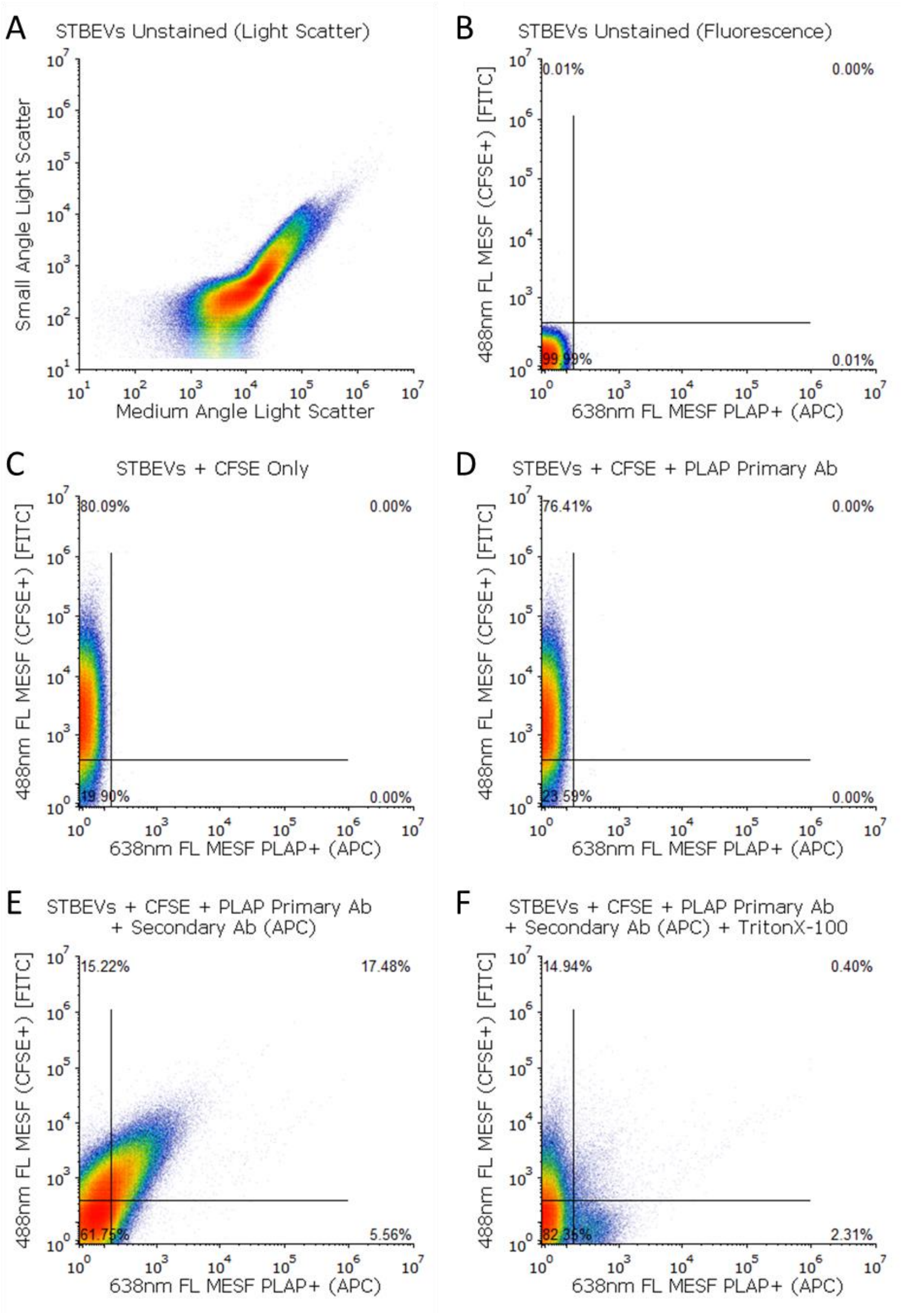
A
STBEVs Unstained (Light Scatter)
Small Angle Light Scatter
Medium Angle Light Scatter
B
STBEVs Unstained (Fluorescence)
0.01%
0.00%
99.99%
0.01%
C
STBEVs + CFSE Only
80.09%
0.00%
19.90%
0.00%
D
STBEVs + CFSE + PLAP Primary Ab
76.41%
0.00%
23.59%
0.00%
E
STBEVs + CFSE + PLAP Primary Ab + Secondary Ab (APC)
15.22%
17.48%
61.75%
5.56%
F
STBEVs + CFSE + PLAP Primary Ab + Secondary Ab (APC) + TritonX-100
14.94%
0.40%
82.35%
2.31%
488nm FL MESF (CFSE+) [FITC]
638nm FL MESF PLAP+ (APC)

**Figure S6**

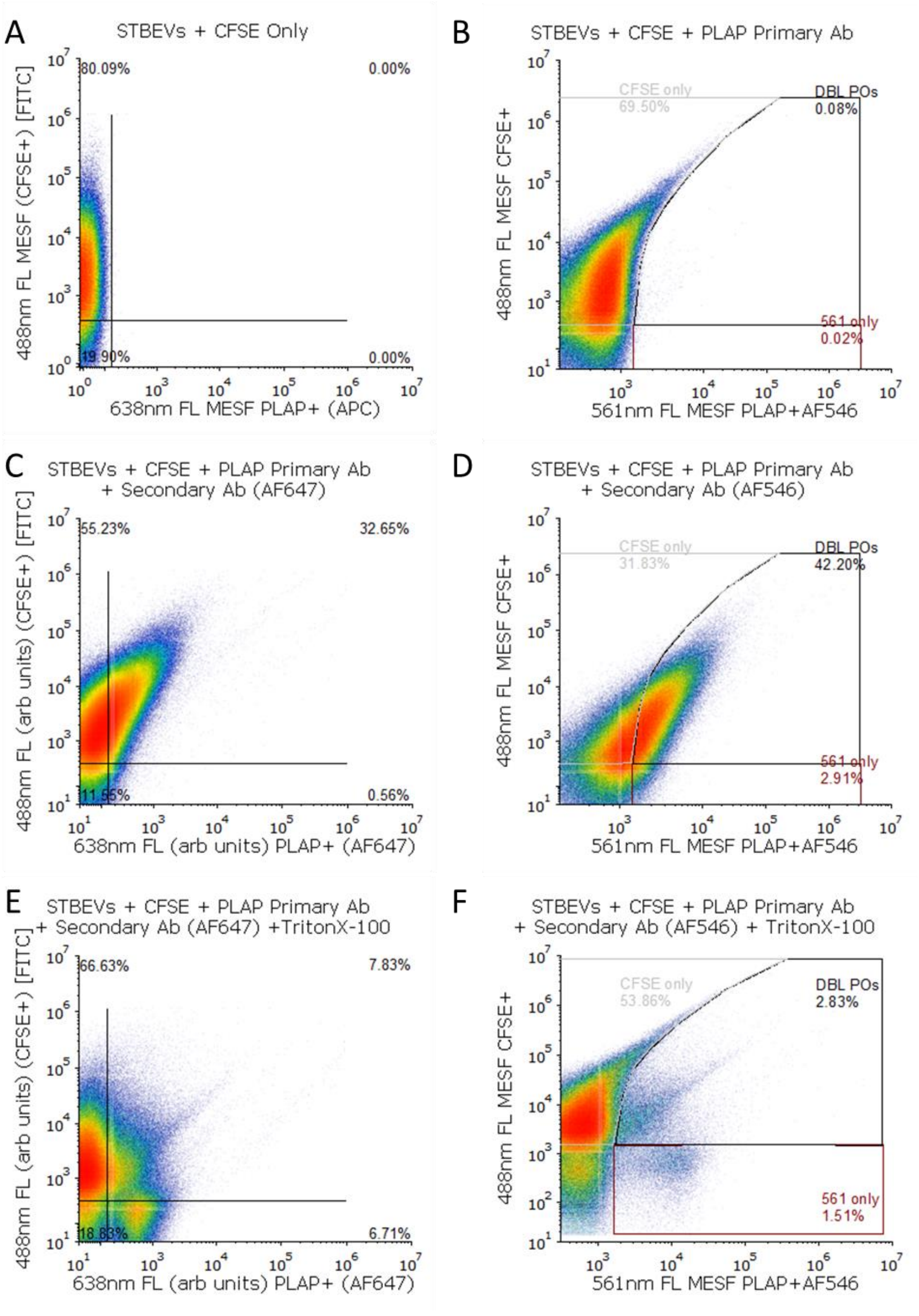

A
STBEVs + CFSE Only
488nm FL MESF (CFSE+) [FITC]
638nm FL MESF PLAP+ (APC)
80.09%
0.00%
19.90%
0.00%
B
STBEVs + CFSE + PLAP Primary Ab
488nm FL MESF CFSE+
561nm FL MESF PLAP+AF546
CFSE only
69.50%
DBL POs
0.08%
561 only
0.02%
C
STBEVs + CFSE + PLAP Primary Ab
+ Secondary Ab (AF647)
488nm FL (arb units) (CFSE+) [FITC]
638nm FL (arb units) PLAP+ (AF647)
55.23%
32.65%
11.55%
0.56%
D
STBEVs + CFSE + PLAP Primary Ab
+ Secondary Ab (AF546)
488nm FL MESF CFSE+
561nm FL MESF PLAP+AF546
CFSE only
31.83%
DBL POs
42.20%
561 only
2.91%
E
STBEVs + CFSE + PLAP Primary Ab
+ Secondary Ab (AF647) +TritonX-100
488nm FL (arb units) (CFSE+) [FITC]
638nm FL (arb units) PLAP+ (AF647)
66.63%
7.83%
18.83%
6.71%
F
STBEVs + CFSE + PLAP Primary Ab
+ Secondary Ab (AF546) + TritonX-100
488nm FL MESF CFSE+
561nm FL MESF PLAP+AF546
CFSE only
53.86%
DBL POs
2.83%
561 only
1.51%

### Whole Western Blotting Membranes

**TSG101 and PLAP**

**Gel: Polyacrylamide 10%**

**Protein Ladder: BLUeye Prestained Protein Ladder (GeneDireX Inc). Red Fluorescence.**

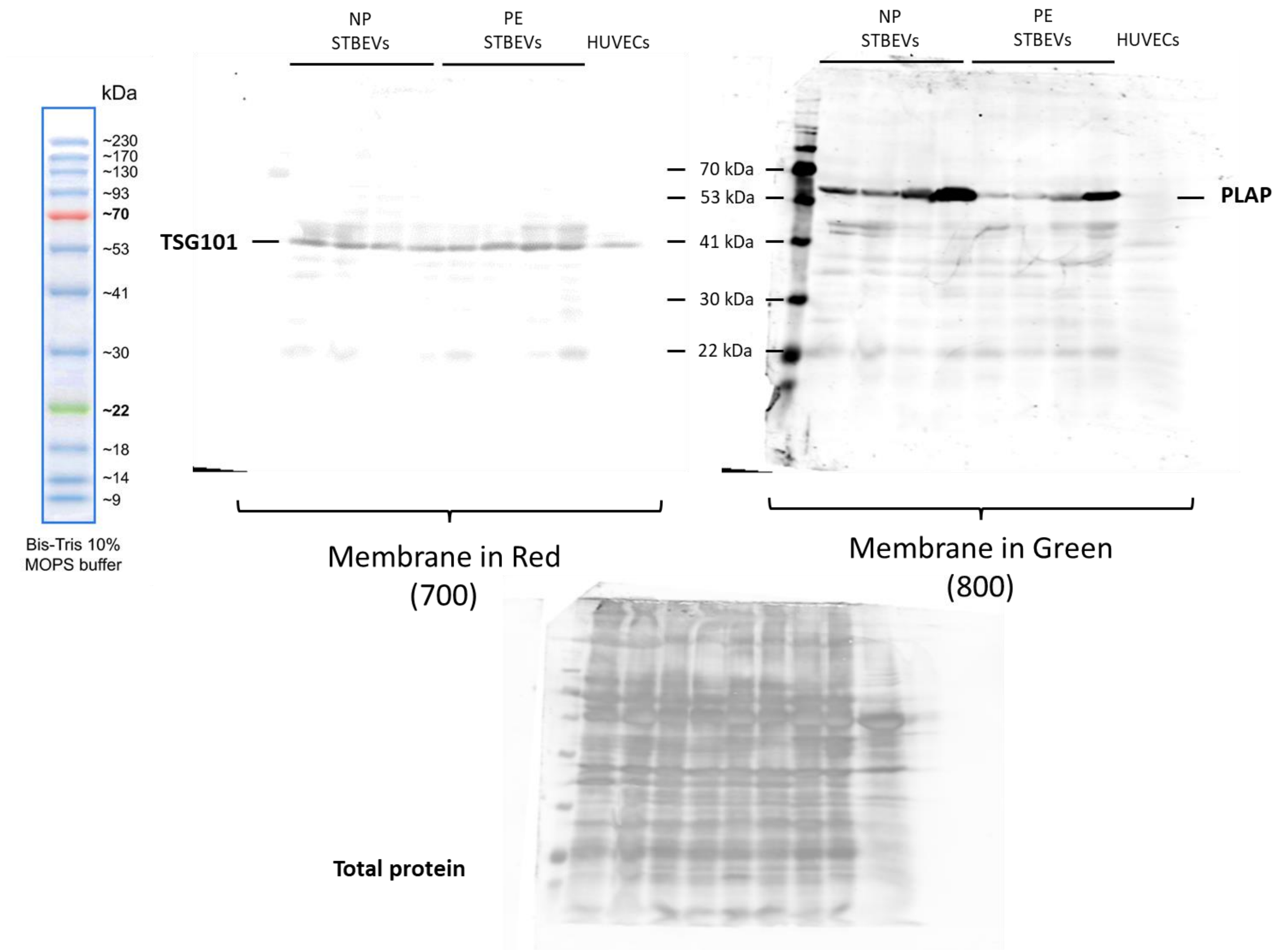


**TSG101**

- Mouse monoclonal antibody, MilliporeSigma #T5701
- Size: ~45 kDa
- 2nd: IRDye™ 800RD-labeled donkey anti-rabbit (green)

**NDOG**

- Mouse monoclonal antibody against PLAP.
- Size: ~70 kDa
- 2nd: IRDye™ 680RD-labeled donkey anti-rabbit (red)

**Cytochrome C**

**Gel: Polyacrylamide 12%**

**Protein Ladder: BLUeye Prestained Protein Ladder (GeneDireX Inc). Red Fluorescence.**

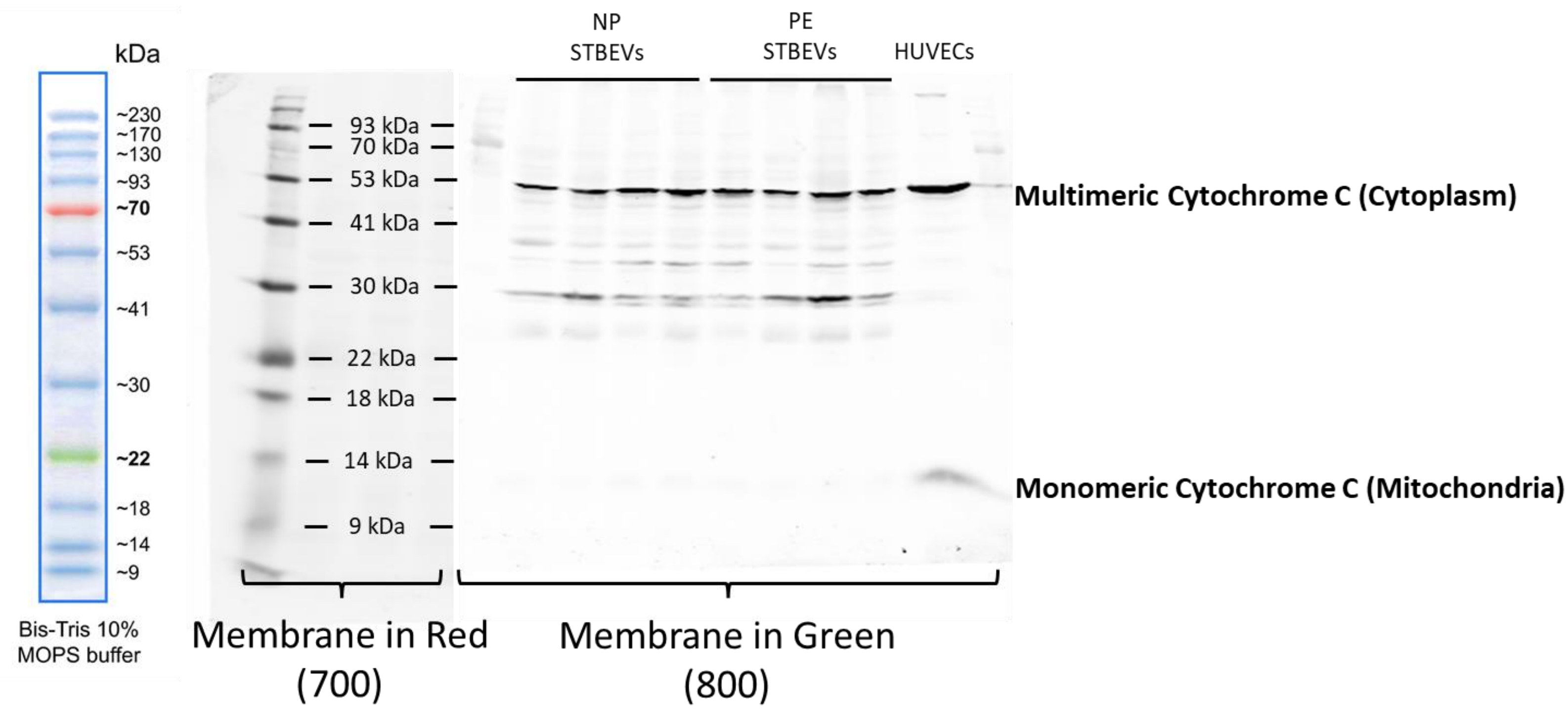


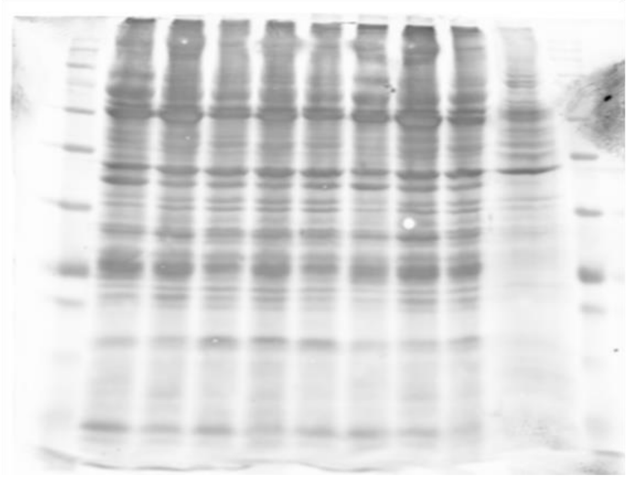

**Total Protein**

**Cytochrome C**

- Mouse monoclonal antibod, BD Bioscience #556433
- Size: ~15 kDa
- 2nd: IRDye™ 800RD-labeled donkey anti-mouse (green)
